# Tunable organic solvent nanofiltration in self-assembled membranes at the sub–1 nm scale

**DOI:** 10.1126/sciadv.abm5899

**Published:** 2022-03-16

**Authors:** Yizhou Zhang, Dahin Kim, Ruiqi Dong, Xunda Feng, Chinedum O. Osuji

**Affiliations:** 1 Key Laboratory of Organic Compound Pollution Control Engineering, Ministry of Education, and School of Environmental and Chemical Engineering, Shanghai University, Shanghai 200444, China.; 2 Department of Chemical and Biomolecular Engineering, University of Pennsylvania, Philadelphia, PA 19104, USA.; 3 Center for Advanced Low-dimension Materials, State Key Laboratory for Modification of Chemical Fibers and Polymer Materials, Donghua University, Shanghai 201620, China.

## Abstract

Organic solvent–stable membranes exhibiting strong selectivity and high permeance have the potential to transform energy utilization in chemical separation processes. A key goal is developing materials with uniform, well-defined pores at the 1-nm scale, with sizes that can be tuned in small increments with high fidelity. Here, we demonstrate a class of organic solvent–stable nanoporous membranes derived from self-assembled liquid crystal mesophases that display such characteristics and elucidate their transport properties. The transport-regulating dimensions are defined by the mesophase geometry and can be controlled in increments of ~0.1 nm by modifying the chemical structure of the mesogen or the composition of the mesophase. The highly ordered nanostructure affords previously unidentified opportunities for the systematic design of organic solvent nanofiltration membranes with tailored selectivity and permeability and for understanding and modeling rejection in nanoscale flows. Hence, these membranes represent progress toward the goal of enabling precise organic solvent nanofiltration.

## Teaser

Self-assembled membranes with uniform pore sizes tunable in 0.1-nm steps have been developed for organic solvent nanofiltration.

## INTRODUCTION

Separation processes in the chemical industry account for approximately 15% of the total global energy consumption (*1*). This high rate of consumption is due to the prevalence of thermal processes in which phase changes are an intrinsic part of the separation, cf. distillation, evaporation, and drying (*2*). Organic solvent nanofiltration (OSN) is an emerging molecular separation technology that features an order of magnitude lower energy spend and is recognized for its potential to substantially alter the landscape in the chemical industry in terms of energy efficiency of separation processes (*3*–*5*). Progress in recent years has delivered working OSN based on solvent-compatible polymers using conventional materials, typically processed through phase inversion, interfacial polymerization, or direct coating of thin-film composite (*4*, *6*, *7*). In these cases, separation occurs primarily due to permselective transport in dense selective layers as opposed to separation by sieving during flow through nanopores. While high-permeability membranes are desired for maximizing process throughput, there exists an intrinsic trade-off between solvent permeability and solute selectivity in OSN (and other) membranes (*8*, *9*). For conventional OSN membranes operating by permeselective transport, this trade-off arises because of the presence of heterogeneous and nonuniform permeation pathways (*10*). The large-scale use of these membranes is often not viable because of their low solvent permeance, e.g., ~1 liter m^−2^ hour^−1^ bar^−1^ for methanol (*4*). In addition, selectivity is challenged by the diminished role of electrostatics in the low-polarity environment of organic solvents compared with aqueous media—the selectivity of aqueous nanofiltration membranes often has a strong contribution from electrostatic interactions between traveling solutes and the membrane (*11*, *12*). In the absence of such a contribution in OSN membranes, effective solute rejection is more heavily reliant on the physical characteristics of the transport-regulating features in the system, viz the pore size and the size distribution in nanoporous membranes, or the size of free volume elements and their distribution in dense membranes.

Recent studies have highlighted the potential of polymeric materials for effective OSN in several contexts. Livingston and co-workers (*13*) proposed a sophisticated yet practical methodology to prepare crumpled, sub–10 nm thickness free-standing polyamide films with high permeance that were incorporated as selective layers in a composite membrane. Alternatively, contorted one-dimensional (1D) ladder-like polymers with intrinsic microporosity (PIMs) leverage inefficient chain packing to create interconnected sub–2 nm volumes and have demonstrated solvent permeance two orders of magnitude higher than Starmem240 commercial membranes (*14*). The rapid structural relaxation of 1D PIMs presents a hindrance to their implementation in ultrathin films. 3D PIMs are more robust in this regard, and a designed 3D polyarylate system with enhanced microporosity achieved a high methanol permeance of ~8 liter m^−2^ hour^−1^ bar^−1^ based on a 20-nm-thick membrane (*15*). Work by Liang *et al.* (*16*) used surface-initiated polymerization of dibromobenzene to generate rigid pores with well-defined structural persistence that efficiently separate ~1.5-nm-size solutes with methanol permeances of ~20 liter m^−2^ hour^−1^ bar^−1^. The preparation of high-permeance (>10 liter m^−2^ hour^−1^ bar^−1^) OSN membranes with sub–1 nm selectivity remains elusive however. Such membranes are highly desired for the separation of low–molar mass solutes (<300 Da) and for solvent purifications. Covalent organic and metal-organic frameworks (COFs and MOFs) offer promise in this regard in their ability to present tailored pores at sub–1 nm–length scales (*17*–*19*). However, the straightforward fabrication of defect-free sheets of such materials over macroscopic areas presents a critical challenge.

Self-assembled lyotropic mesophases based on small molecules (molar mass, ~500 Da) are characterized by thermodynamically prescribed morphologies with well-defined structural periodicities (*20*–*22*). Appropriately prepared, these materials can provide transport-regulating features at ~1-nm-length scales associated with the lamellar, cylindrical, or gyroid motifs (*23*–*25*), and their potential in membrane applications is highlighted in prior research (*26*–*29*). The ordered nanostructure circumvents the challenges associated with the dispersity of transport-regulating features in conventional membranes highlighted earlier and offers the compelling possibility of overcoming the selectivity-permeability trade-off seen in current OSN membranes. To first order, the transport-regulating feature size of a self-assembled mesophase is determined by the size of the constituent molecular species and its concentration in the lyotropic system. The utilization of self-assembled systems for OSN therefore offers the attractive possibility of fine-tuning the cutoff characteristics of membranes by appropriately altering the size of the molecular building blocks and/or their concentration in the phase. For example, work by Gin and co-workers (*30*, *31*) polymerized bicontinuous cubic (Q_I_) mesophases from various surfactant molecules to ensemble membranes with pore sizes between 0.75 and 0.96 nm.

While self-assembled materials hold significant potential for improved OSN performance, we are not aware of any work to date implementing self-assembled nanostructures for OSN in the 1-nm regime. This is likely due to the significant challenges associated with doing so—apart from the difficulty associated with maintaining membrane stability in organic solvents with different polarities, the difficulty of preserving self-assembled nanostructures in thin films (*32*–*34*) and the potential need for nanostructural alignment to ensure good permeance (*35*–*37*) represent significant obstacles in the fabrication of practical OSN membranes with ordered nanostructure. A recently developed strategy addresses these challenges however. The internally cross-linked cylinders from a direct lyotropic hexagonal (H_I_) mesophase present an attractive medium for nanofiltration, because of the bicontinuous nature of the solvent transport and the well-defined ~1-nm slit-like pores provided by the space between the surfaces of nearest-neighbor cylinders uniformly oriented in the plane of the film (*38*, *39*).

Here, we introduce a method for the fabrication of cross-linked hexagonal mesophase-based membranes for OSN and present data on their solute rejection and permeability characteristics. We explore a series of mesophases prepared using different alkyl chain length surfactants and cross-linking chemistries with H_I_ domain spacing tailored from 3.1 to 3.9 nm. The resulting membranes exhibit systematic variation in pore size that is manifested accordingly in their transport characteristics. In comparison to several recently reported polymeric OSN membranes, the mesophase-derived membranes reported here are highly ordered and display higher solvent permeabilities. These nanostructured membranes operate effectively in sieving-based separation, as demonstrated by solute rejection experiments in different organic solvents. The self-assembled materials explored here may provide a new paradigm for membranes with precisely tunable nanostructure for demanding OSN applications.

## RESULTS AND DISCUSSION

**Fig. 1.**
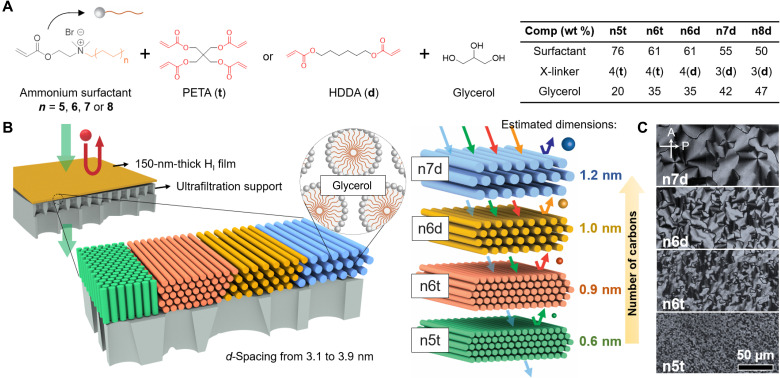
Schematic illustration of self-assembled OSN membranes with pore sizes tuned by tailoring the lyotropic mesophase chemistry. (**A**) Chemical structure of the polymerizable surfactants and other components (PETA or HDDA cross-linkers; glycerol containing 10% water) of the mesophase. The specific mesophase compositions are tabulated. The alkyl chain length is varied by changing *n* (*n* = 5, 6, 7, and 8) to target nanostructured membranes with different pore sizes. (**B**) Illustration of different H_I_ thin films with unit cell dimensions d_100_ ranging from 3.1 to 3.9 nm prepared atop ultrafiltration supports. The cylinders of the mesophase may orient vertically (cf. green cylinders) or horizontally. For parallel cylinders, the transport-regulating dimension is the distance between neighboring cylinder surfaces, and this ranges from ~0.6 to 1.2 nm for the four systems studied in detail. (**C**) Magnified POM micrographs of the polymerized H_I_ thin films reveal the preservation of their original textures from the lyotropic mesophase. The orientations of the polarizer (P) and the analyzer (A) are indicated by the arrows.

We used a series of polymerizable surfactants that feature a hydrophobic ethyl acrylate group and alkyl tail jointly linked to a hydrophilic quaternary ammonium head group. These molecules were synthesized using a one-step Menshutkin reaction targeting different carbon chain lengths (*38*). Mesophases are prepared by mixing the surfactants with glycerol [containing 10 weight (wt %) water] and a selected cross-linker [either pentaerythritol tetraacrylate (PETA; t) or hexanediol diacrylate (HDDA; d)] with designated weight fractions, shown in Fig. 1. Four polymerizable surfactants were used with differing alkyl chain lengths corresponding to 18 (n8), 16 (n7), 14 (n6), and 12 (n5) methylene groups in the hydrophobic tail, where the *n* stands for repeating ethyl unit, as shown in Fig. 1A. Each surfactant was screened to identify appropriate compositions that provided stable hexagonal lyotropic mesophases at room temperature. All four species formed H_I_ mesophases with unit cell dimensions ranging from ~3 to 4 nm. Here, we designate the mesophases according to the type of surfactant molecule and the cross-linker presented within, as n5t, n6t, n6d, n7d, and n8d, respectively. Figure 1B provides the schematic of the proposed OSN membranes and the associated transport-regulating dimensions templated by lyotropic mesophases.

Glycerol is a low-volatility liquid (vapor pressure <1 torr at 20°C). It is used as the medium for lyotropic assembly to facilitate structure retention during solution-based processing by avoiding changes in composition due to evaporative loss. The components of the mesophase were dissolved in ethyl acetate at 10 wt % (vapor pressure ~70 torr at 20°C) to yield a low-viscosity solution. The solution was spin coated on various substrates, followed by ultraviolet (UV)–initiated cross-linking in a nitrogen atmosphere to produce solid films. The mesophase structure in fabricated thin films was examined by polarized optical microscopy (POM) coupled with grazing incidence small-angle x-ray scattering (GISAXS) on different substrates. Comparison of the optical textures of the mesophase in its initial lyotropic gel state versus those in the cross-linked gel reveals excellent retention of the hexagonal structure after UV-induced cross-linking (see fig. S1). Magnified POM images of the polymerized mesophases are shown in Fig. 1C.

**Fig. 2.**
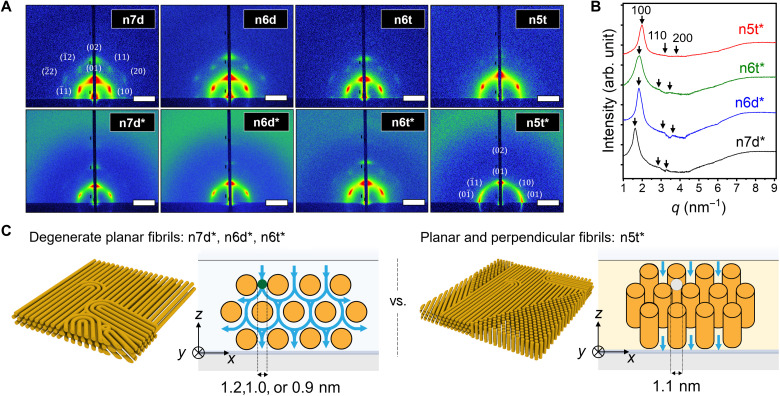
Structural characterization and dimensional analysis of different H_I_ thin films cast from 10 wt % mesophase solutions. (**A**) 2D GISAXS data from samples prepared by spin coating and cross-linking different mesophase thin films atop either bare silicon wafers (top row) or on PVP (bottom row, noted with *). The GISAXS data reflect the existence of hexagonal mesophases with different orientations of the cylindrical nanostructures. Scale bars, 2 nm^−1^. (**B**) 1D GISAXS data acquired from H_I_ coating on PVP-covered surfaces. (**C**) Schematic showing the estimated critical dimensions for transport within the mesophases as inferred from the x-ray data for n7d*, n6d*, n6t*, and n5t* systems. The calculations are based on the assumption that the bromide anions dissociate from the surfactant molecule.

GISAXS was conducted for thin films prepared by spin coating 10 wt % mesophase solutions onto polished silicon wafers and on wafers coated with a thin layer (~3 μm) of polyvinyl pyrrolidone (PVP). PVP surfaces that were used as its orthogonal solubility relative to the cross-linked mesophase makes it a good candidate for a sacrificial layer for membrane fabrication on porous supports (discussed below). Data are shown in Fig. 2 (A and B). For mesophases containing surfactants with hydrophobic tails shorter than 18 carbons, the Bragg reflections from 1D GISAXS data (generated by azimuthal integration of the 2D data) from the PVP-H_I_ composites occur at scattering vector ratios of 1:√3:√4. This is consistent with high-fidelity retention of the H_I_ mesophase structure after cross-linking (Fig. 2B). However, the cross-linking of the n8d mesophase was accompanied by disruption of the mesophase order, as manifested by a marked change in optical textures observed by POM (fig. S2). GISAXS data (fig. S3) indicate that the n8d system undergoes a transformation to a lamellar structure during cross-linking, and so this system was not extensively studied.

The 2D GISAXS data provide important information regarding the orientation of the nanostructures produced by spin coating and cross-linking the mesophases. The hexagonal symmetry observed for n7d, n6d, and n6t on both silicon and PVP surfaces indicates that the cross-linked cylinders in the system are oriented with their long axes parallel to the plane of the substrate. By contrast, for n5t, while such a planar configuration was observed on silicon substrates, a mixture of planar and perpendicular, or vertical, cylinders was observed for films prepared on PVP. The existence of vertically oriented cylinders is implied by the concentration of scattering intensity along the equatorial line of the scattering plane. The observed mixture of planar and perpendicular cylinders was found for n5t samples prepared from a broad range of surfactant concentrations (fig. S4). We surmise that the effect of the PVP on the morphology is more pronounced for this 12-carbon alkyl surfactant system, relative to the others. One possibility is that the planar configuration is metastable and kinetically dictated during the rapid assembly during spin coating, following which the low mobility of longer-chain surfactant mesophases precluded rearrangement (*40*, *41*). We consider it more likely, however, that the observed differences are related to differences in the energetics of the substrate interactions, which can vary considerably with composition and molar mass of the constituent species. For the planar arrangements, the orientation of the cylinders in the plane of the film is not constrained, as evidenced by the lower intensity of the off-meridional (10) reflections relative to the meridional (01) reflection. Furthermore, the discrete azimuthal intensity variation indicates that there is a preferred orientation of the hexagonal lattice in the films, with the close-packed planes parallel to the film surface.

The display of planar versus perpendicular orientations of the cylinders has important implications for selective transport in the fabricated films. The transport-limiting dimension for solutes traveling through the film along its thickness (the *z* axis) is larger for perpendicular cylinders than it is for planar cylinders (fig. S5). The ratio between the two is the diameter of the circular void at the center of a triangle connecting the centers of three nearest-neighbor cylinders relative to the distance between surfaces of any pair of nearest-neighbor cylinders. This ratio *P* = 2δ/*S_x_* = (4/3 − ξ)/[(4/3)^1/2^ − ξ], where $\xi ={\left(8\varphi /\sqrt{3}\pi \right)}^{1/2}$ for a system with volume fraction of cross-linked cylinders is given by φ. For example, with φ = 0.50, *P* is 1.6. From the d_100_ spacings provided by GISAXS, we calculate the transport-limiting dimensions associated with the planar and mixed planar/perpendicular orientations of the cylinders. For n5t, the transport-limiting dimension is 2δ, associated with travel parallel to the cylinder axes, whereas for the other mesophases, it is the smaller critical dimension, *S_x_*, associated with travel orthogonal to the cylinders. The transport-limiting dimensions ranged from ~0.9 to 1.2 nm, as shown in Fig. 2C (additional details in fig. S5). In addition, this dimension is readily tunable by adjusting the surfactant weight fractions in the lyotropic phase (additional mesophase with structural analysis shown in figs. S6 and S7) and was tailored in fine increments of ~0.1 nm (fig. S8). The interplay of composition with molecular structure provides a rich design space for creating fit-for-purpose membranes with tailored dimensions.

**Fig. 3.**
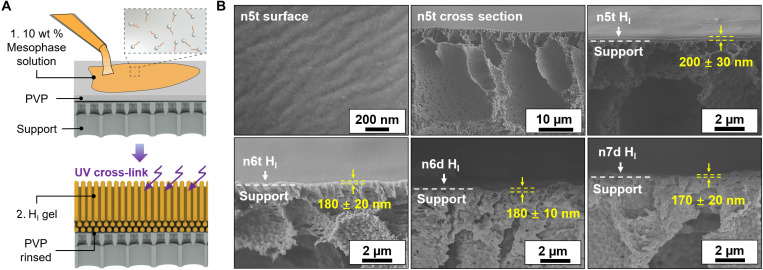
Schematic illustration of the fabrication of thin mesophase–derived membranes and images of their resulting structure. (**A**) Schematic describing the self-assembled H_I_ thin film composite membrane fabrication process. (**B**) Top view scanning electron microscopy (SEM) images of a cross-linked n5t H_I_ mesophase after thoroughly rinsing to remove the PVP sacrificial layer, and cross-sectional views of n5t, n6t, n6d, and n7d membranes atop PVDF supports after removal of PVP sacrificial layers. Error bars correspond to 95% confidence interval from a minimum of three measurements.

Thin (i.e., less than ~200 nm) membranes were prepared for the characterization of transport properties by spin casting thin layers of the lyotropic mesophases on sacrificial layer–coated ultrafiltration supports. The sacrificial layer prevents the infiltration of the mesophase solution into the support membrane during casting. The fabrication process is illustrated in Fig. 3. A PVP film with a thickness of ~3 μm was coated on polyvinylidene fluoride (PVDF) ultrafiltration membranes by spin coating. Subsequently, mesophase solutions in ethyl acetate were directly spin coated on the composite; PVP is insoluble in ethyl acetate and is therefore preventing entry of the mesophase into the PVDF support. The mesophase thin film was kept quiescently under ambient conditions for ~1 min to facilitate solvent evaporation, following which it was photopolymerized by exposure to UV light. The resulting composite membranes were permeated with deionized water (DI) water in a stirred dead-end filtration cell for ~12 hours, until steady permeances were recorded, indicating that the sacrificial layer had been fully removed. Membranes were kept in the filtration cell for the weeks-long duration of their testing and were thereafter removed for imaging by scanning electron microscopy (SEM). Images are shown in Fig. 3B. Micrographs of high-magnification views with false color overlays delineating the selective layers are shown in fig. S9. In addition, no delamination of the selective layer from the underlying support was observed during this period.

**Fig. 4.**
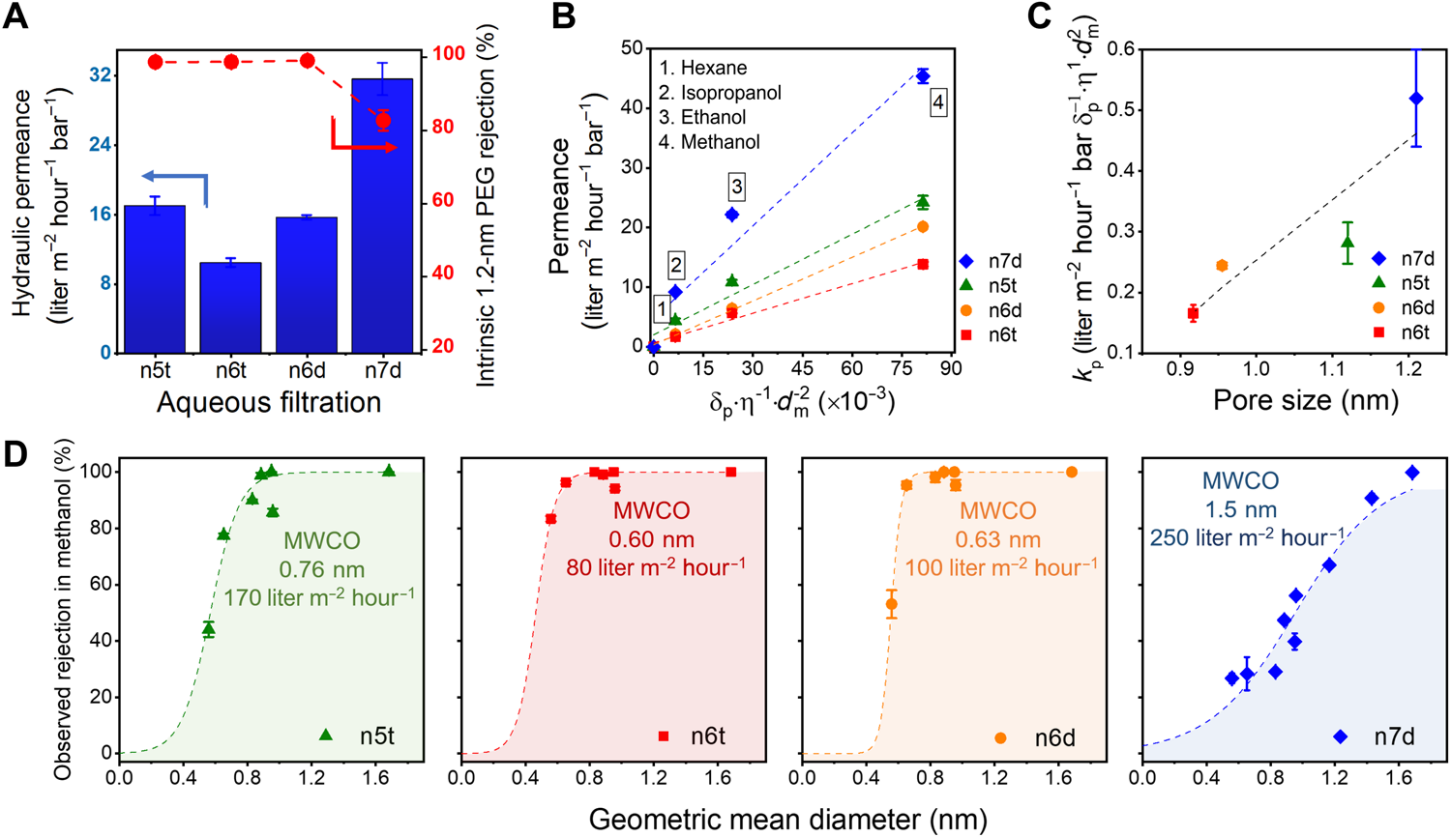
Nanofiltration performance of the H_I_-templated membranes in aqueous and organic environments. (**A**) Survey of transport performance among four H_I_ membranes in DI water. The single solute rejections of 600 g mol^−1^ PEG molecule are displayed with the hydraulic permeance. (**B**) Pure solvent permeances follow a phenomenological model composed of solubility parameter, viscosity, and solvent molar diameter. The dashed lines represent the linear regressions that derive the proportionality constant *k*_p_ proportional to the calculated membrane pore sizes shown in (**C**). (**D**) Observed solute rejection performance measured by permeating different dye molecules dissolved in methanol across the membrane at a consistent transmembrane pressure of 5 bars. The observed MWCOs are located at 0.6, 0.63, 0.76, and 1.5 nm for n6t, n6d, n5t, and n7d membranes, respectively. The curves are fitted with a sigmoidal model. Error bars correspond to 95% confidence interval from a minimum of three replicates. Where absent, the error bars are smaller than the symbol size. The methanol flux recorded during the neutral red rejection is listed with the MWCO curves.

The transport properties of the membranes were first characterized using aqueous solutions (dielectric constant, ε = 80.2) containing ~1.2-nm hydrodynamic diameter polyethylene glycol (600 g mol^−1^ PEG) (*42*–*44*). The PEG has no significant or persistent shape anisotropy. Hence, the rejection data with PEG provide a realistic representation of the minimum dimension of the slit-like pores in between cylinders. This situation contrasts with that of highly anisotropic solutes that could, in principle, transit through the slit-like pores despite having a molar mass above the nominal cutoff (*39*). The measured hydraulic permeance is in good agreement with theoretical estimates made for ordered planar oriented cylinders (*45*) and a hypothetical mixture of perpendicular and planar orientations (*46*), as shown in fig. S10. The observed rejections were corrected for the effects of concentration polarization (~0.3 to 3% change) using the correlation developed by Zeman and Zydney (*47*). Complete rejection was observed for membranes based on mesophases with calculated limiting dimensions lower than 1.2 nm (assuming complete ammonium bromide dissociation), as shown in Fig. 4A. The n7d membranes, with a transport-regulating dimension estimated at 1.2 nm, demonstrated a rejection of 83%. The transport-regulating dimension is estimated on the simplifying assumption that the Br^−^ counterion is a fixed part of the mobile phase. It is likely that the degree to which Br^−^ is part of the stationary versus the mobile phase is a strong function of solvent polarity, and we anticipate that the effective pore size may be modestly larger in high-polarity media such as water that favors dissociation of the counterion from the quaternary ammonium co-ion. Nevertheless, the observed rejections, under convective transport conditions, are unambiguous regarding the regime of solute sizes that can be effectively filtered by the membranes.

Before performing organic solvent filtration experiments, we examined the stability of the membranes on exposure to various solvents by GISAXS measurements using n6d as a model system. Cross-linked films on silicon wafers were immersed in various solvents and then vacuum dried for subsequent structural characterization. GISAXS data show a preservation of structure, as manifested by retention of hexagonally arranged Bragg spots with a d_100_ spacing of ~3.4 nm, as detailed in fig. S11. These data indicate that the membranes retain their nanostructure in the presence of the organic solvents considered here. Note that the choice of solvents was constrained not by the stability of the cross-linked mesophase but by that of the support membrane.

Organic solvent permeation experiments were conducted in dead-end filtration cells at ~20°C. Data are shown in Fig. 4B. Permeance was derived from the linear regression of solvent flux to the applied pressure detailed in fig. S12. The solvent permeances and solute rejections did not change measurably over the weeks-long durations of the permeation tests. This steady transport performance indicates that the nanostructures within the membrane, and the composition overall, are highly stable under the test conditions used here. The highest permeance recorded was 45 liter m^−2^ hour^−1^ bar^−1^ for passage of methanol through an n7d membrane. The same membrane showed a ~5× reduction in permeance for isopropanol, while hexane exhibited no measurable permeation, even at a high transmembrane pressure of 28 bars.

While the standard continuum models for transport in nanoporous membranes invoke viscosity as the only fluid property that affects permeance, for nanofiltration membranes and specifically for OSN, permeance is affected by factors beyond viscosity. A phenomenological model invoking the diameter of the solvent molecule, the solubility parameter, and the dynamic viscosity was developed by Buekenhoudt *et al*. (*48*) to account for permeance of various solvents in porous nanofiltration (NF) and ultrafiltration (UF) ceramic membranes and applied by Livingston and co-workers (*13*) successfully for polyamide thin films. The model implicitly seeks to account for solvent-membrane interactions, the effects of excluded volume on solvent species transport, and momentum diffusion, although there are several caveats given the correlation among the three representative parameters. As advanced by Livingston and co-workers (*13*), based on correlations highlighted by Buekenhoudt *et al*. (*48*), the model provides a transport parameter, *k*_p_, and posits that permeance scales linearly with the solvent parameter $\delta_{p}\eta^{-1}d_{m}^{-2}$, i.e., that permeance *P* is proportional to *k*_p_ as

$$P=k_{p}(\frac{\delta_{p}}{\eta^{1}d_{m}^{2}})$$ (1)

where δ_p_ is the polarity factor of the solubility parameter, η is the solvent viscosity, and *d*_m_ is the solvent molecular diameter. Our solvent permeation results are well described by the model as shown by the data for four different solvents in each of the four membranes (Fig. 4B). Furthermore, the constant of proportionality, *k*_p_, shows a linear relationship with pore size (Fig. 4C). The significance of this pore size dependence is unclear. Buekenhoudt *et al*. (*48*) considered water flux–normalized data and remarked that pore size may play an indirect role in the water permeance–normalized proportionality factor, *C*, contained therein, and defined as (*P*η)/(*P*_w_η_w_) − 1 ∝ *C* (δ_tot_/δ_tot, w_ − 1), with this factor decreasing exponentially for larger pores. However, the proportionality depended also on the surface chemistry of the commercially produced Inopor membranes, which varied even for membranes with the same nominal pore size, and so the dependence of the proportionality factor to pore size could not be unambiguously delineated. Furthermore, the pore sizes of the membranes were broadly distributed, which could smear out a clear role regarding pore size. Here, our membranes provide precise, small changes in transport-regulating dimensions that are narrowly distributed. Our analysis of water flux–normalized data excluding the hexane permeance (fig. S13) suggests that the Buekenhoudt proportionality factor *C* scales linearly with pore size for our membranes. We anticipate that these membranes may prove useful in generating data against which existing concepts for nanoscale transport may be evaluated, or using which new concepts may be developed.

Rejection experiments were performed in methanol (ε = 32.7) using a constant transmembrane pressure of 5 bars and a variety of molecular solutes with sizes in the range of 0.5 to 2 nm, at a concentration of 50 μM, while the permeate was collected after the steady state was achieved. The membranes displayed distinct rejection characteristics. As shown in Fig. 4D, the molecular weight cutoff (MWCO) varied from ~0.6 to 1.5 nm across the four types of membranes considered. This range of sizes corresponds to ~290 to 800 g mol^−1^ in terms of molar mass (space-filling models for each solute are displayed in fig. S14). The methanol flux recorded during the nanofiltration of neutral red (289 g mol^−1^) is indicated on each MWCO curve. Membranes n6d and n6t demonstrate similar selectivity with only a minor difference in their MWCOs (320 versus 290 g mol^−1^, respectively). This is expected, given the single angstrom difference in their lattice constants, with d_100_ spacings of 3.4 and 3.5 nm. Meanwhile, the n8d membrane with the largest *d*-spacing of 3.9 nm exhibited a cutoff at 1.5 nm and correspondingly displayed the highest solvent flux, 250 liter m^−2^ hour^−1^, in neutral red solution. For n5t membranes, the large MWCO of 350 g mol^−1^ reflects the larger transport-limiting dimension in this system, despite having the smallest *d*-spacing of 3.1 nm. As discussed earlier, this is due to the presence of some vertically oriented cylinders, which result in larger pores being available for transport than the case for all planar cylinders.

**Fig. 5.**
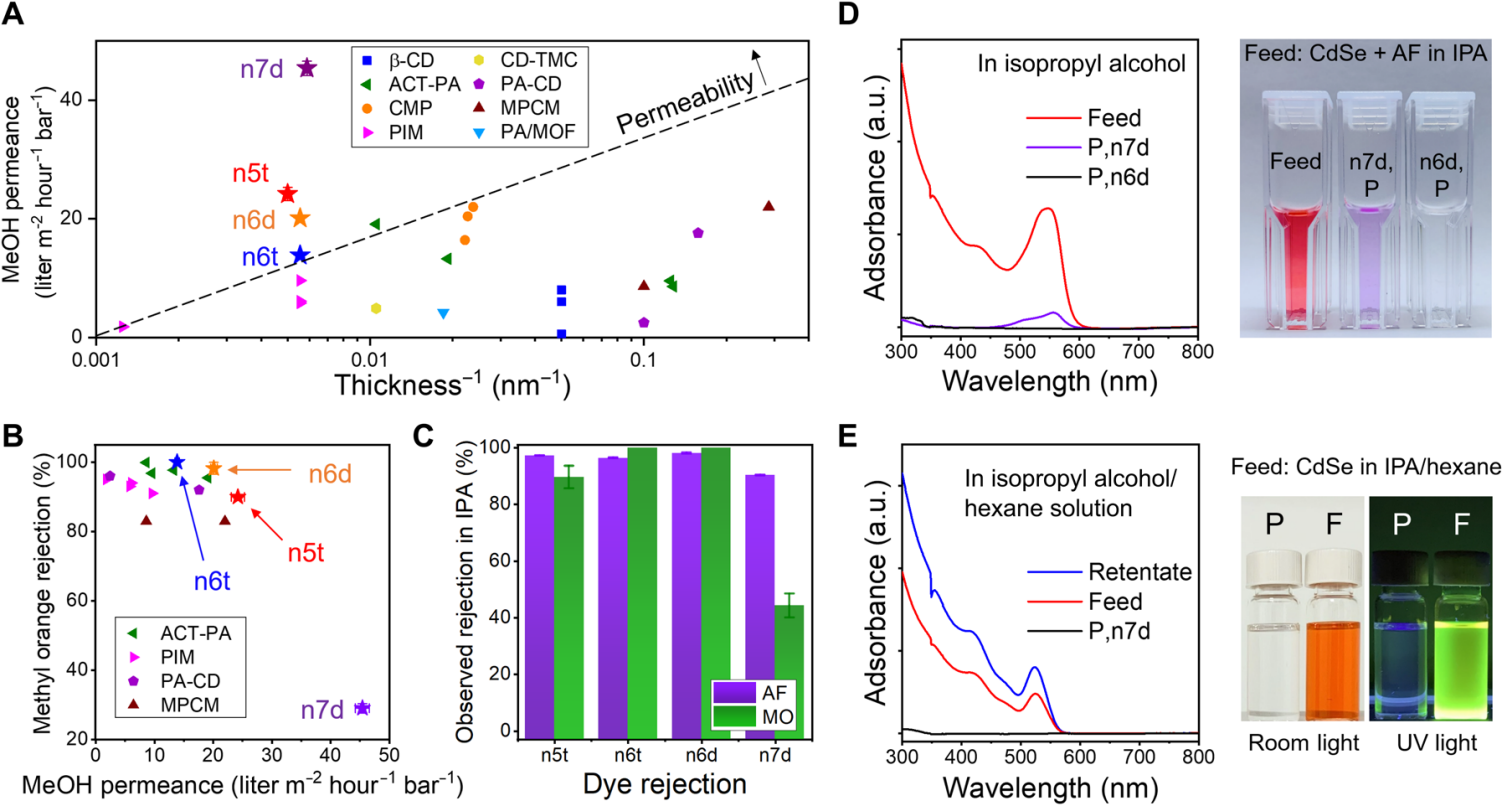
Single solute and dual, or competitive, OSN performance. (**A**) Methanol permeance as a function of membrane thickness for H_I_ membranes compared with other reported polymeric OSN membranes including polyamides [solvent activated polyamide (ACT-PA) and PA/MOF] (*13*, *51*), PIMs (*15*), rigid conjugated microporous polymers (CMP) (*16*), β–cyclodextrin-terephthaloyl chloride (β-CD) (*52*), cyclodextrin–trimesoyl chloride (CD-TMC) (*53*), polyamide-cyclodextrin (PA-CD) (*54*), and molecularly porous polyamide (MPCM) (*55*). (**B**) Comparison of the methyl orange rejection and methanol permeance for the H_I_ membranes to other materials in literature. (**C**) Single solute rejection data of different mesophase-templated membranes for acid fuchsin (AF) and methyl orange (MO) dissolved in IPA. (**D**) UV-vis spectrum and photograph for competitive separation of CdSe nanoparticle and AF dissolved in IPA by n6d and n7d membranes. (**E**) UV-vis data and photographs of the luminescent quantum dots demonstrating the n7d solute rejection in a solvent mixture of 5:5 (by volume) is IPA to hexane solution. Error bars are propagated 95% confidence limits derived from multiple measurements. Where absent, the error bars are smaller than the symbol size. a.u., arbitrary units. F, feed.

Figure 5A provides a summary of methanol permeance versus the reciprocal membrane thickness for our mesophase-derived membranes, along with data for several recently reported polymeric OSN membranes. The current work validates the concept of uniformly nanostructured OSN membranes and demonstrates the tunability inherent in the mesophase self-assembly. Nonetheless, these membranes exhibit higher permeance and, in the majority of cases, also higher permeability, relative to recently reported systems. A more detailed performance comparison is provided by considering solute rejection as a function of solvent (methanol) permeance, shown in Fig. 5B. The data highlight the improved solvent permeance and the superior permeability (fig. S15) at high rejection levels. Note that in the case of n7d, the data are uncorrected for the effects of concentration polarization, which negatively affect rejection under high flux conditions. We examined solute rejection performance in isopropyl alcohol (IPA; ε = 19.9) at a consistent solvent flux ~10 liter m^−2^ hour^−1^. As shown in Fig. 5C, the n7d membrane exhibits a higher rejection to both acid fuchsin (AF) and methyl orange (MO) in the IPA rejection experiment than in methanol and demonstrates an improved MWCO of ~0.9 nm (590 g mol^−1^). Associated UV–visible (UV-vis) spectra and images are shown in fig. S16. The reduction in MWCO in IPA versus methanol is likely a result of concentration polarization under the higher flux conditions during methanol operation. Enhanced ion pairing in IPA relative to methanol due to dielectric permittivity differences may also contribute to this effect.

The filtration of particulate species and competitive filtration of particulate and molecular species were investigated using 2.7-nm-diameter CdSe quantum dots (EM images in fig. S17) and mixtures of these dots with molecular dyes, respectively. Filtration of CdSe suspended in IPA using n6d and n7d membranes showed complete rejection of CdSe (fig. S18) as expected, given the <1-nm MWCO based on rejection data for these membranes when confronted with molecular dyes in IPA (Fig. 5C). Data for competitive rejection experiments with CdSe in the presence of AF in IPA are shown in Fig. 5D. Both membranes rejected CdSe completely. The n6d membrane rejected AF completely, but for n7d, the rejection was 90%. In a second experiment using a smaller dye, methylene blue (MB), CdSe was again completely rejected, whereas the rejection of MB was 85 and 60% for n6d and n7d, respectively (fig. S19). These results highlight the efficacy of these membranes for selective solute separations.

We also examined the filtration of CdSe quantum dots dispersed in a 1:1 volume mixture of IPA and hexane using n7d membranes. Results are shown in Fig. 5E. The quantum dots were completely rejected as expected. In addition however, analysis of the permeate showed evidence of differential transport of IPA and hexane through the membrane. CdSe photoluminescence (PL) is sensitive to the nature of the surrounding solvent, and in particular, PL is reduced in polar solvents such as IPA, relative to hexane (*49*). Results show enhanced PL in the retentate (fig. S20) relative to the original feed, indicating that hexane enrichment occurred in the feed, and IPA enrichment in the permeate. Differential transport is expected on the basis of the differences in solubility parameters of the solvents and molecular size. The extent of the enrichment could not be quantitatively determined from the PL data however. While these results are preliminary, they are consistent with the expectation that differential solvent transport will occur in these systems and highlight potential utility for these membranes in solvent separations.

Our work demonstrates a strategy to prepare self-assembled membranes for high-performance OSN in the 1-nm regime. Fabrication of thin mesophase–derived membranes was enabled by a facile solution process with the resulting cross-linked materials exhibiting stability in a range of organic solvents, and the membranes demonstrated stable filtration performance. Variation of the size (alkyl chain length) of the self-assembling constituent species provides a reliable handle for tuning the transport-limiting dimensions of the derived membranes, with MWCO points observed ranging from 0.6 to 1.5 nm. Solvent transport is well described by a phenomenological model invoking viscosity, solubility parameter, and solvent molecular size. Differential solvent transport was demonstrated, highlighting the potential for solvent separations. These membranes stand out for their high permeabilities relative to a broad range of amorphous polymeric membranes. We anticipate that the approach demonstrated here can be extended to other mesogens, perhaps tailored for OSN or other separations of interest. Furthermore, we expect that the current solution-based spin coating method can be migrated to roll-to-roll solution processing using blade or dip coating for the membrane fabrication at a larger scale. This work may facilitate the development of new energy-efficient membrane applications, especially for organic solvent applications that request exquisite sieving performance, such as biopharmaceutical purification, heterogeneous membrane reactors, or functional nanoparticle remediation.

## MATERIALS AND METHODS

### Synthesis of the surfactant molecules

All chemicals used in this study were purchased from Sigma-Aldrich and used as received unless otherwise noted. Glycerol (≥99.5%, Fisher Scientific, maximum water content 0.5%) was diluted with 10 wt % DI (*R*= 18 megohm·cm) to prepare 90 wt % glycerol stock solution. Cross-linkers, including both PETA and HDDA (Alfa Aesar), were doped with 1 wt % photoinitiator 2-methoxy-2-phenylacetophenone (Acros Organics) before use. The cationic surfactants with variable alkyl chain lengths, 2-(acryloyloxy)ethyl dodecyl dimethyl ammonium bromide (AEDDAB, n5), 2-(acryloyloxy)ethyl tetradecyl dimethyl ammonium bromide (AETDAB, n6), 2-(acryloyloxy)ethyl hexadecyl dimethyl ammonium bromide (AEHDAB, n7), and 2-(acryloyloxy)ethyl octadecyl dimethyl ammonium bromide (AEODAB, n8) were synthesized through a modified one-step Menshukin reaction. Specifically, in a typical reaction, 1.00 mol of 2-(dimethylamino)ethyl acrylate was mixed with 1.05 mol of bromoalkanes (1-bromododecane, 1-bromotetradecane, 1-bromohexadecane, or 1-bromooctadecane) and 0.01 mol of hydroquinone in a 500-ml round-bottom flask with a Teflon stir bar. The mixture was further diluted with 200 ml of binary solvent composed of 50/50 (v/v) tetrahydrofuran and acetonitrile to form a homogeneous solution and was stirred at 45°C for 48 hours. After 48 hours, the solution was allowed to cool to room temperature. Subsequently, the solid product was precipitated in excessive anhydrous diethyl ether three times, followed by drying in vacuo overnight.

### Mesophase formulation and membrane casting

Mesophase thin films with different limiting spacings were prepared in a fume hood operated in an air-conditioned environment with temperature regulated between 16° and 23°C, and relative humidity varied between 10 and 40%. Namely, distinct ternary mixtures that self-assemble to H_I_ lyotropic mesophases were identified and cast in thin films for further characterizations. n5t (composed by 76 wt % AEDDAB, 4 wt % PETA, and 20 wt % glycerol), n6t (61 wt % AETDAB, 4 wt % PETA, and 35 wt % glycerol), n6d (61 wt % AETDAB, 4 wt % PETA, and 35 wt % glycerol), n7d (55 wt % AEHDAB, 3 wt % HDDA, and 42 wt % glycerol), and n8d (50 wt % AEHDAB, 3 wt % HDDA, and 47 wt % glycerol) were dissolved in ethyl acetate with predetermined concentrations. Subsequently, individual casting solutions were then filtered through a 0.2-μm polytetrafluoroethylene syringe filter (Fischer Brand) before use. In this manner, different H_I_ thin films with similar self-assembled structures but various domain spacings were fabricated through spin coating the precursor solutions atop different substrates, including microscope glass slides, (100) silicon substrate, and silicon substrates covered with a thin layer of PVP at a consistent spin speed of 2000 revolutions per minute (rpm) for 1 min, and let sit in ambient condition for an extra 1 min. To prepare the PVP casting substrate, a 12 wt % solution of PVP (360 kg mol^−1^) dissolved in ethanol was spin coated atop pieces of the silicon substrate at 3000 rpm for 3 min. Subsequently, the silicon wafer with the PVP solution was transferred into a convection oven heated at 80°C for 30 min for complete solvent evaporation.

Nanofiltration membranes were fabricated as thin film composites atop commercial ultrafiltration supports. PVDF ultrafiltration membranes (Synder V6 PVDF with MWCO of 500 kg mol^−1^) were purchased from the vendor. Membranes were rinsed in ethanol and spin coated with a thin layer of the sacrificial thin film by 12 wt % PVP (360 kg mol^−1^) from its ethanol solution at 3000 rpm for 5 min. The 10 wt % mesophase solutions dissolved in ethyl acetate were subsequently cast atop the ultrafiltration supports using the same mesophase spin coating protocol described above.

After the H_I_ mesophase thin film casting, samples were immediately transferred into an enclosed nitrogen atmosphere. The photoinitiated cross-linking of films was conducted by illuminating a focused UV beam (100-W SunSpot SM with three types of UV rays covering a range of wavelengths from ~275 to 450 nm) 8 cm above the substrate surface for 25 min. To examine the resiliency of H_I_ nanostructure in different solvent environments, some n6d silicon wafer–supported thin films were immersed in different organic solvents for 30 min and were subsequently dried in high vacuum for further structural analysis.

### Structural characterizations

The birefringence textures of the liquid crystal mesophases were analyzed using a Zeiss Axiovert 200M inverted microscope. A charge-coupled device camera photographed the corresponding micrographs for different mesophases before and after the photoinitiated cross-linking. Meanwhile, a JEOL 7500F field emission scanned electron microscope high-resolution SEM characterized the nanoscale morphology of the H_I_ membranes. For surface analysis, membranes were sectioned into 1-cm × 1-cm pieces using a razor blade. Vacuum-dried membranes were cryofractured in liquid nitrogen for cross-sectional imaging. Samples were mounted on standard stages and were sputter coated with ~3-nm iridium before loading into the microscope chamber. Micrographs were captured at working distances from 6 to 8 mm with a constant accelerating voltage of 5 kV.

2D GISAXS data were collected by a Xeuss 2.0 from Xenocs at the Dual Source and Environmental X-ray Scattering facility operated by the Laboratory for Research on the Structure of Matter at the University of Pennsylvania. The scattering data were acquired from a GeniX3D Cu source (λ = 1.54 Å) at a constant sample to detector distance of 55 cm, covering accessible scattering vectors (*q*) 0.016 to 1.02 Å^−1^. Samples were mounted on a GISAXS stage with the incident angle to the x-ray maintained from 0.15 to 0.25. After the scattering experiment, 1D integrated data were further processed by using the Foxtrot software package with the scattering intensity (*I*) versus *q* = 4πsin(θ)/λ, where the scattering angle is 2θ. Silver behenate was the corresponding calibration standard. Since the perpendicular *q* projection *q_z_*′ is much greater than the critical angle *q*_c_ (~0.01 Å^−1^), it was estimated that *q_z_* ~ *q_z_*′ in the final 1D plot.

### Synthesis of CdSe nanoparticles

The synthesis of the oleic acid (OA) capped CdSe nanoparticles was performed following a previously reported hot injection procedure (*50*). Specifically, CdO (0.79 mmol) was dissolved in a mixture of OA (3.9 mmol) and 1-octadecene (14 ml) in a three-neck round bottom flask heated at 100°C to remove oxygen. The mixture was heated to 180°C until a homogeneous solution was acquired, followed by elevating the temperature to 250°C. At the same time, another solution was prepared by dissolving tri-*n*-octylphosphine (0.27 mmol) and Se (0.26 mmol) in 1-octadecene (2 ml). The CdSe nanoparticle was acquired by injecting the Se-containing solution to the Cd-containing solution at 250°C, and the mixture was allowed to cool down to room temperature after the injection. The product was then dissolved in toluene and precipitated in methanol. The nanoparticles were then dispersed in hexane or isopropanol solution and were used immediately in the following transport experiments after the synthesis. The nanoparticles were imaged using a JEOL F200 field emission transmission electron microscope at an accelerating voltage of 200 kV.

### Transport experiments

The solvent permeation and solute rejection performance for different mesophase membranes were characterized using a dead-end 50-ml Amicon stirred cell (UFSC05001) equipped with a customized circular stainless steel surface area reducer and a stir bar. The reducer exposes an active surface area of 2.4 cm^2^ available for solvent permeation, and the cell was stirred at 400 rpm to reduce the concentration polarization during the rejection experiments. Membranes were placed in the stirred cells supported by the polypropylene (PP)/polyethylene (PE) nonwoven mat with their self-assembled layer facing the feed solution. Compressed nitrogen was provided by a dual-gauge regulator delivering transmembrane pressure up to 80 psi. After the membrane fabrication, the composite membranes were rinsed thoroughly with DI water for ~12 hours to detach the PVP layer sandwiched between the ultrafiltration support and the thin film. The solvent permeance experiments (including DI water, methanol, ethanol, isopropanol, and hexane) were conducted in an air-conditioned environment with room temperature that varied between 16° and 20°C. The permeance of various solvents was generally measured following a sequence of DI water, hexane, methanol, ethanol, and isopropanol using transmembrane pressure from ~2 to 75 psi. Membranes were stored in DI water between each solvent permeance characterizations. In the case of the hexane permeation experiment, membranes were placed in an HP4750 stirred cell installed with a 25-mm membrane area reducer connected to a high-pressure hose supplying transmembrane pressure at 400 psi. The permeated solvent was weighed every 5 min at different pressures to calculate the solvent permeance.

The single solute rejection experiment in aqueous solution was performed using 600 g mol^−1^ PEG (with polydispersity index Đ < 1.2; Polymer Source Inc.) molecule dissolved in DI water with a concentration of 1 g liter^−1^ as standard. The polymer solution was permeated through the membranes at a consistent flux ~12 liter m^−2^ hour^−1^, and at least 5 ml of permeate solution was collected during each rejection experiment. The polymer concentration at the permeate side was analyzed by a modified Dragendorff reagent method based on a Cary 100 UV-vis spectrophotometer that calculates the analyte abundance using Beer-Lambert’s law. The MWCO curves in methanol were determined by permeating dye molecules of different sizes with a concentration of 50 μM through the membrane at a consistent transmembrane pressure of 75 psi. Before each organic solvent rejection experiment, the membranes were first cleaned with saturated NaNO_3_ methanol solution, followed by rinsing with excessive DI water and the pure organic solvent from the prospective rejection experiment.

Most solute rejection experiment in isopropanol was determined by permeating the single solute solution containing 50 μM dye molecules, 5 μM CdSe, or mixtures of dyes and CdSe at the concentrations as mentioned above through the membranes with a consistent flux of ~10 liter m^−2^ hour^−1^. In the solute competitive solute rejection experiment with the AF, a higher CdSe concentration of 50 μM was involved. During the OSN experiment, the sample collection was started once a steady-state permeate concentration was observed, and at least 5 ml of permeate solution was collected for further analysis using the UV-vis spectrophotometer. In the mixture solvent permeation, using a consistent transmembrane pressure drop at 75 psi, an equal volume of isopropanol and hexane prepared 50 μM CdSe feed solution. The retentate solution and the concentrated solution (concentration normalized to retentate) containing equal-volume hexane and isopropanol were further analyzed by an Edinburgh Instruments FLS1000 fluorescence spectrometer that excited the samples using a 365-nm monochromated Xe arc lamp.

## Supplementary Materials

### This PDF file includes:

Sections S1 and S2 Figs. S1 to S20 References
