# Supplementary material for: Tunable organic solvent nanofiltration in self-assembled membranes at the sub–1 nm scale
Source: Sci Adv. 2022 Mar 16;8(11):eabm5899. doi: 10.1126/sciadv.abm5899 (PMC8926336; doi:10.1126/sciadv.abm5899)
Supplement: Supplementary file 1 — Sections S1 and S2 Figs. S1 to S20 References [file sciadv.abm5899_sm.pdf]

Science Advances
AAAS

Supplementary Materials for

Tunable organic solvent nanofiltration in self-assembled membranes at the sub–1 nm scale

Yizhou Zhang, Dahin Kim, Ruiqi Dong, Xunda Feng, Chinedum O. Osuji*

*Corresponding author. Email: cosuji@seas.upenn.edu

Published 16 March 2022, *Sci. Adv.* **8**, eabm5899 (2022)
DOI: 10.1126/sciadv.abm5899

This PDF file includes:

Sections S1 and S2
Figs. S1 to S20
References

Section S1. Calculations of the limiting dimensions

H_I nanofiltration membranes function by an ordered array of hexagonally close-packed nanofibrils with well-defined center-to-center spacings from d = 3.1 to 3.9-nm. The prior study suggests the swelling by water is negligible. Here because the polymeric nanostructures from lyotropic self-assembled mesophases were cross-linked and anchored by covalent bonds, we assume the influence of solvent swelling is negligible. The solvent continuous nanochannel schematic listing the nomenclature of the geometric parameters is shown in Figure S5 below. Note the dissociation of the counterion Br^- from NH_4Br depends on the solution dielectric constant, ε. Hence, the dissociated Br^- may be presented as a part of the solvent continuum in aqueous solutions; alternatively, a larger diameter cylinder can be expected if there is no ionic dissociation. In turn, 2 cylinder volume fractions φ for each mesophase are approximated. The limiting dimension is derived from the below calculations:

$$l = \frac{2d}{3} \tag{S.1}$$

$$\delta = l - r_f \tag{S.2}$$

$$t = l \cdot \sin\left(\frac{\pi}{3}\right) \tag{S.3}$$

$$2\delta = \mathrm{d}\left[\frac{4}{3} - \left(\frac{8\varphi}{\sqrt{3}\pi}\right)^{\frac{1}{2}}\right] \tag{S.4}$$

$$S_x = 2t - 2r_f = \mathrm{d}\left[\sqrt{\frac{4}{3}} - \left(\frac{8\varphi}{\sqrt{3}\pi}\right)^{\frac{1}{2}}\right] \tag{S.5}$$

$$S_y = 3l - 2r_f = \mathrm{d}\left[2 - \left(\frac{8\varphi}{\sqrt{3}\pi}\right)^{\frac{1}{2}}\right] \tag{S.6}$$

Section S2. Calculations for the transport characterization

Hydraulic Permeance Calculation

For ordered cylinders with low Reynolds fluid flow perpendicular to the long axis, the dimensionless hydraulic permeability $k_{=}^{*}$ of can be approximated as a function of solid volume fraction φ (*45*),

$$k_{=}^{*} = \frac{2\sqrt{2}}{9\varphi}(1 - \sqrt{\frac{4\varphi}{\pi}})^{5/2} \qquad (S.7)$$

Similarly, the dimensionless hydraulic permeability $k_{\parallel}^{*}$ of fluid flow parallel to the square array of fibers is (*46*):

$$k_{\parallel}^{*} = \frac{-\ln(\varphi) - 1.476 + 2\varphi - 0.5\varphi^{2} - O(\varphi^{4})}{4\varphi} \qquad (S.8)$$

These calculations lead to the Darcy permeability from the dimensionless permeability factor k^{*} derived above.

$$k = k^{*} \cdot r_{f}^{2} \qquad (S.9)$$

From the above correlation, the hydraulic permeability *P* is estimated by accounting for the water viscosity μ that is 0.0011 Pa·s at 16°C:

$$P = \frac{k}{\mu} \qquad (S.10)$$

Where the *P* is directly related to the hydraulic permeance by factors of membrane thickness δ and tortuosity τ,

$$L_{p} = \frac{P}{\delta} \qquad (S.11)$$

When there are mixed cylinder orientations (*i.e.*, parallel cylinders followed with perpendicular cylinders) across the membrane cross-section, the associated hydraulic permeance L_p is estimated with flow units in series,

$$\frac{1}{L_{p}} = \frac{1}{L_{p,1}} + \frac{1}{L_{p,2}} + \cdots + \frac{1}{L_{p,n}} \qquad (S.12)$$

The Calculation for Transport Characterization

The observed solute rejection R_o was directly calculated by comparing the solute concentration of the permeate to the feed. In the equation below, C_p is the permeate concentration, and C_f is the feed concentration,

$$R_o(\%) = \left(1 - \frac{C_p}{C_f}\right) \times 100\% \tag{S.13}$$

In the aqueous solution rejection (PEG) experiment, the intrinsic solute rejection R_a most accurately reflects the nanochannel sieving property. It was calculated by correlating the observed rejection of the effect of concentration polarization by an equation of the volumetric water flux J_w to the mass transfer coefficient k. Since the J_w/k are always smaller than 0.3, there was no severe concentration polarization in aqueous solution experiments (*47*).

$$R_a(\%) = 1 - R_o + R_o \exp\left(\frac{J_w}{k}\right) \tag{S.14}$$

The concentration polarization correction was between 0.3 to 3% through the entire PEG rejection experiment. The mass transfer coefficient is estimated based on the Colton-Smith empirical correlation defined by a function of projected stir bar radius r, solute diffusion coefficient D_i, solvent kinematic viscosity v, and the rotational speed ω of the magnetic stirring bar,

$$\frac{kr}{D_i} = 0.285 \cdot \left(\frac{v}{D_i}\right)^{\frac{1}{3}} \cdot \left(\frac{\omega r^2}{v}\right)^{0.567} \tag{S.15}$$

Here, the D_i values for polyethylene oxide molecules are retrieved from the literature. For other solutes, the D_i is approximated using the Stoke-Einstein equation,

$$D_i = \frac{k_B T}{6\pi r_s} \tag{S.16}$$

In order to describe the rejection profile of H_I templated membranes, a logistic function is employed to fit the sigmoidal-like progression of the molecular weight cut-off curve. By corresponding the solute diameter estimated from the Chem3D software package to the observed rejection, the fitting parameters β and γ best describing the rejection profile were derived (*56*),

$$R_a(\%) = \left(\frac{1}{1 + e^{-\gamma d_s + \beta}}\right) \times 100\% \tag{S.17}$$

Supplementary Figures

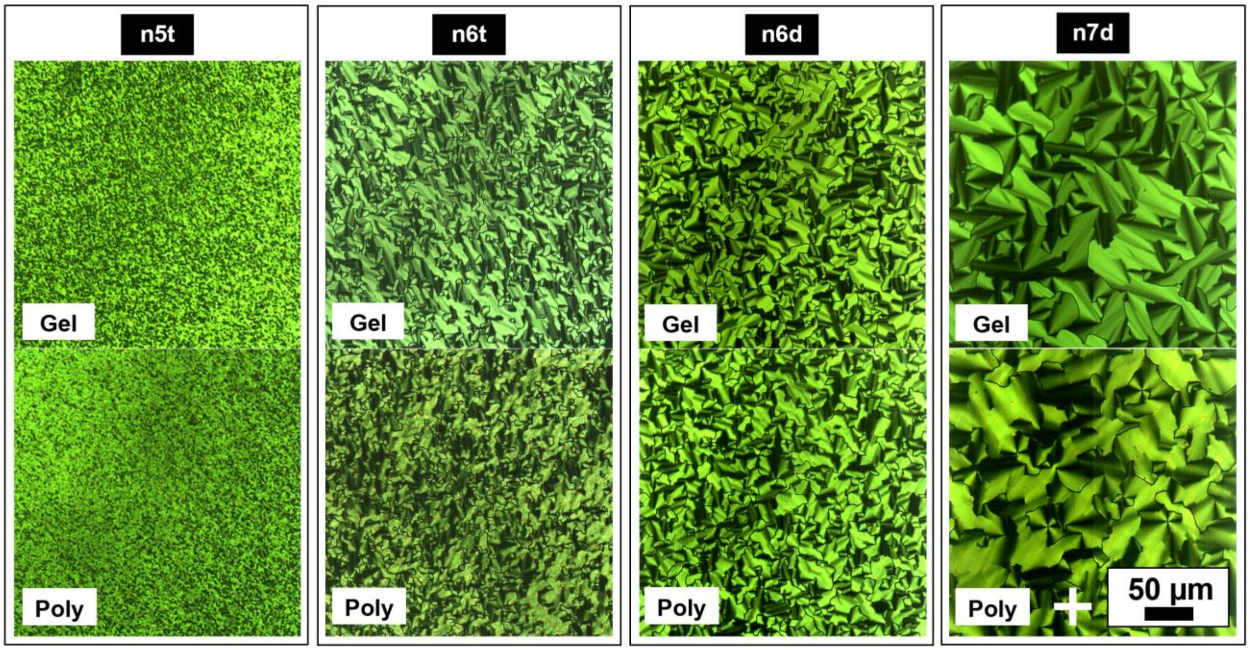

Fig. S1. Polarized optical micrographs (POM) displaying the high-fidelity retention of the mesophase textures. Micrographs were taken before and after the photo-induced polymerization for n5t, n6t, n6d, and n7d in thin-film. 20 wt.% mesophase solutions were spin-coated on pre-cleaned glass slides to prepare ~ 500 nm thick films. Consistent liquid crystal textures were observed without the emergence of optical inhomogeneities in the developed birefringence domains. Gel samples were prepared from ~20 wt.% solutions.

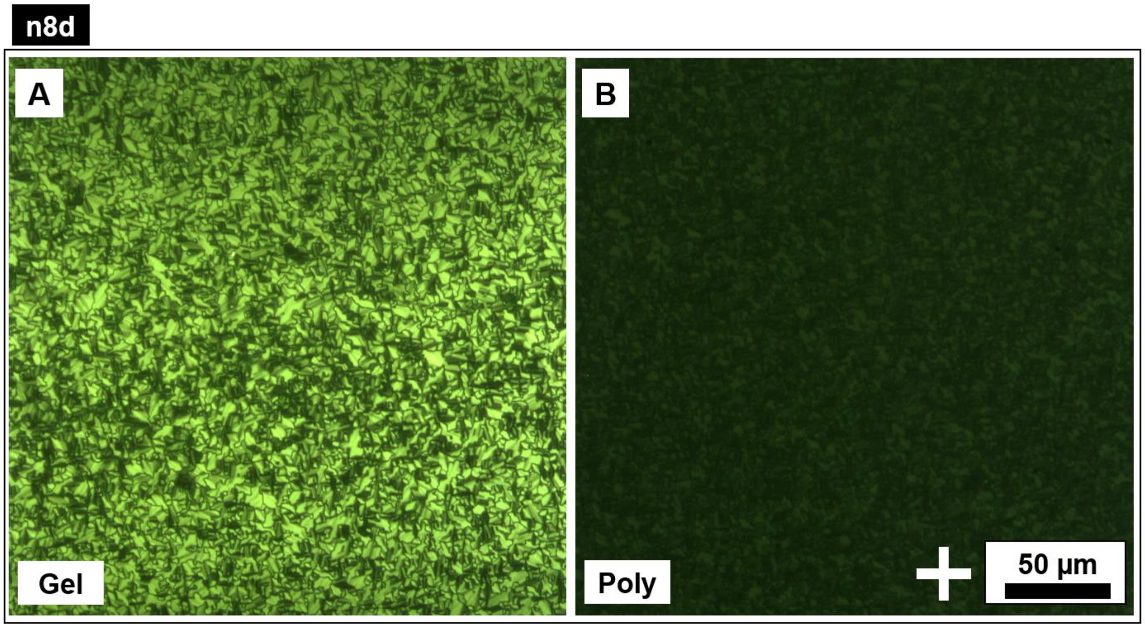

Fig. S2. POM images showing the disruption of the original mesophase texture after the polymerization of n8d mesophase. The micrograph was captured from a thin-film cast on a pre-cleaned glass slide. The fading of birefringence texture in the cross-linked sample indicates potential phase transformation.

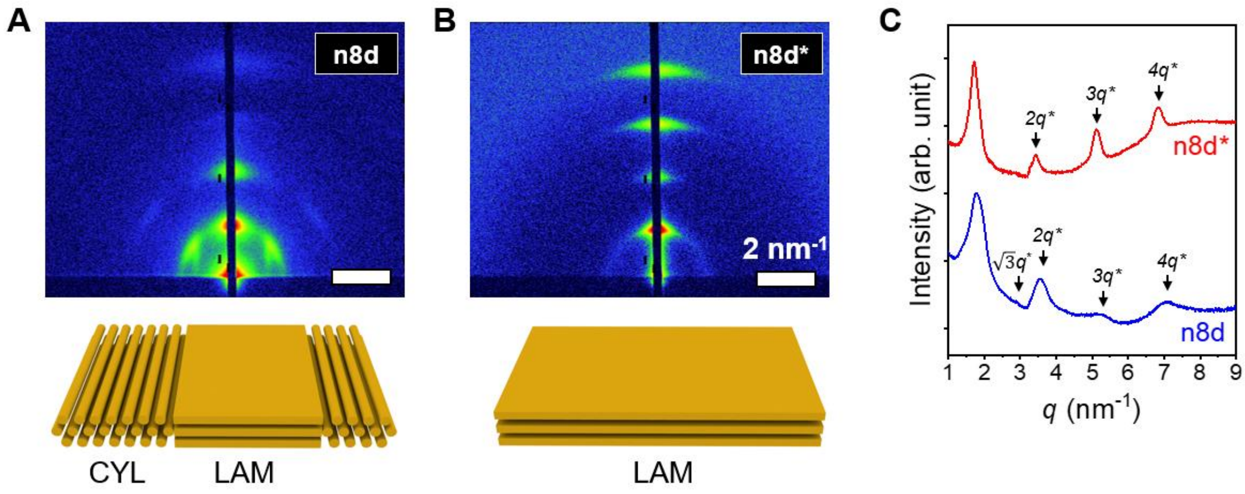

Fig. S3. Grazing-incidence small-angle X-ray scattering (GISAXS) data of the n8d thin-film prepared through the direct solution coating of the precursor solution on silicon substrates. The polymerized nanostructures were analyzed for thin-films (**A**) prepared on an unmodified substrate and (**B**) cast on poly(vinylpyrrolidone) modified surface. Subsequently, the 1D SAXS data was calculated from 2D images shown in (**C**). The X-ray data suggest the phase transformation emerges during the polymerization of n8d mesophase. The peak spacing ratio 1: √3: √4: 3: 4 in the polymerized film evidenced the co-existence of the lamellar and hexagonal close-packed nanostructure. Meanwhile, for the thin-film polymerized on PVP modified surface, the spacing ratio 1: 2: 3: 4 characteristic for the lamellar nanostructure is observed.

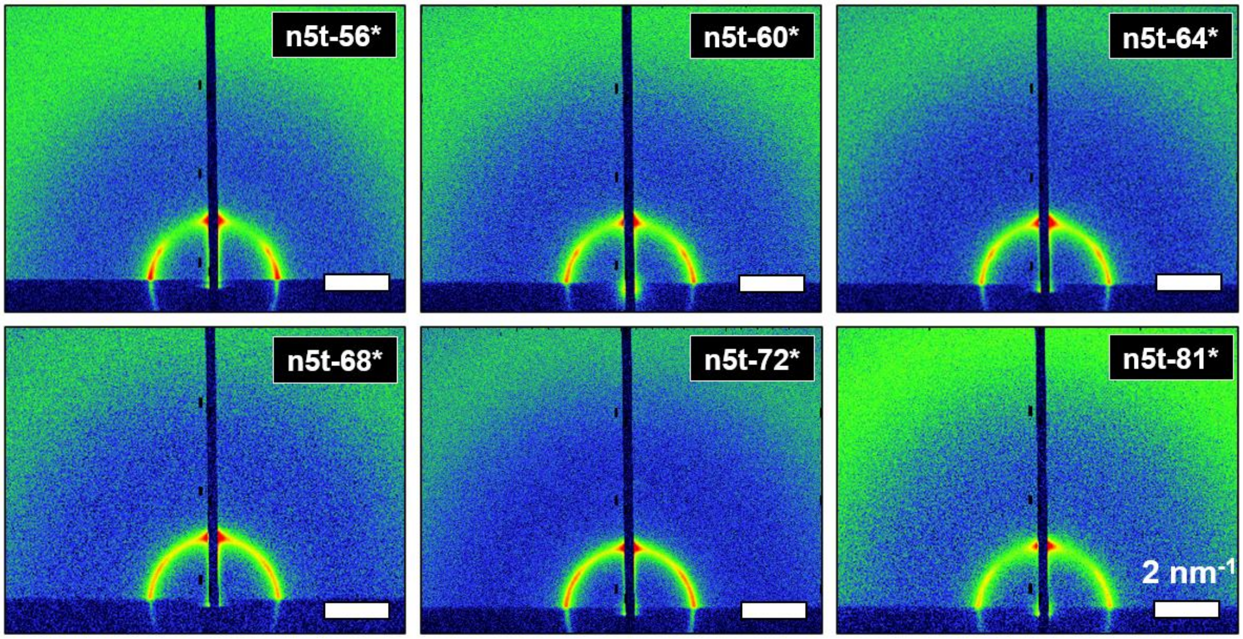

Fig. S4. GISAXS patterns of the cross-linked n5t mesophases initiated from different surfactant concentrations. Mesophases were cast with concentrations as indicated (81 to 56 wt.%) atop of PVP modified substrate. The concentration of the cross-linker is kept at 4wt%, but the glycerol content was adjusted accordingly. The anisotropic pattern is mostly indiscernible from different mesophases examined.

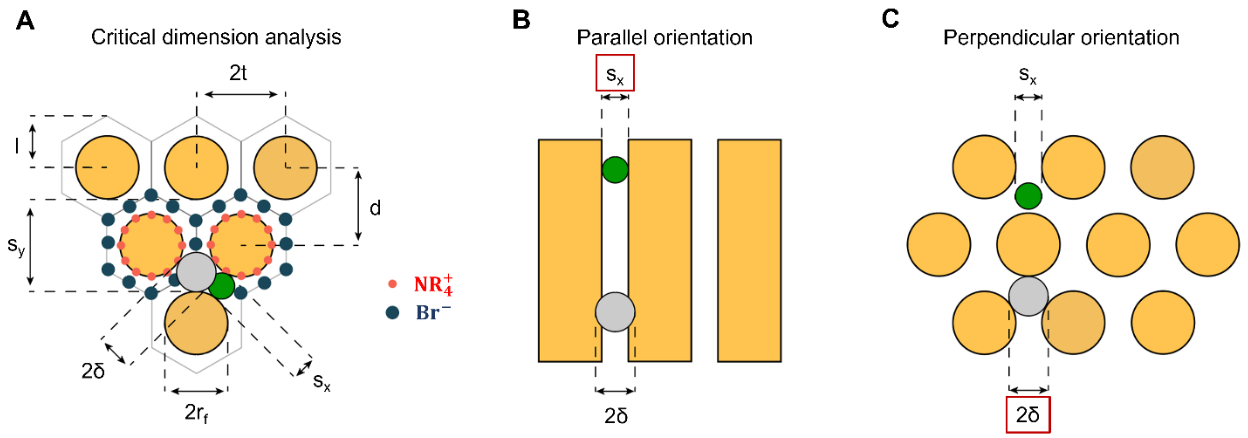

D

High ε solvent (Anions in solvent continuum)					
Mesophase	Cylinder fraction (% by volume)	*d*-spacing (nm)	$2r_f$ (nm)	s_x (nm)	2δ (nm)
n5t*	64.4	3.1	3.02	0.563	1.12
n6t*	53.3	3.4	3.00	0.917	1.52
n6d*	52.9	3.5	3.09	0.955	1.58
n7d*	48.4	3.9	3.29	1.21	1.91

E

Low ε solvent (Anions in solid matrix)					
Mesophase	Cylinder fraction (% by volume)	*d*-spacing (nm)	$2r_f$ (nm)	s_x (nm)	2δ (nm)
n5t*	79.5	3.1	3.35	0.227	0.781
n6t*	65.7	3.4	3.34	0.582	1.19
n6d*	65.3	3.5	3.43	0.610	1.24
n7d*	59.7	3.9	3.65	0.850	1.55

Fig. S5. Schematic for the critical dimension of the hexagonally packed nanofibrils available for nanofiltration. Data are tabulated. The geometrical spacing calculations are estimated in solvent environments with different dielectric constants. The bromide ion is assumed to dissociate from the fibril matrix in solvent with a high dielectric constant or bind with the matrix in solvent with a low dielectric constant, and hence the different volume fractions. The grey particle represents the limiting dimension available for solvent flow along the axial direction, and the green particle is the confining dimension in case of the solvent flow perpendicular to the cylinders.

A

Additional mesophase compositions				
Mesophase	Surfactant (% by weight)	X-linker (% by weight)	X-linker type	Glycerol (% by weight)
n6d-81	81	4	HDDA	15
n7d-72	72	3	HDDA	25
n7t-55	55	3	PETA	42
n7t-72	72	3	PETA	25

B

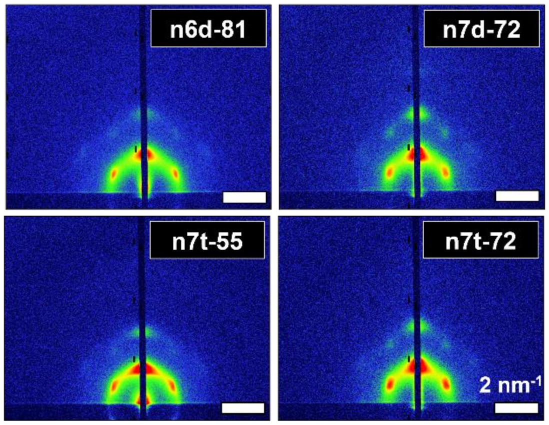

C

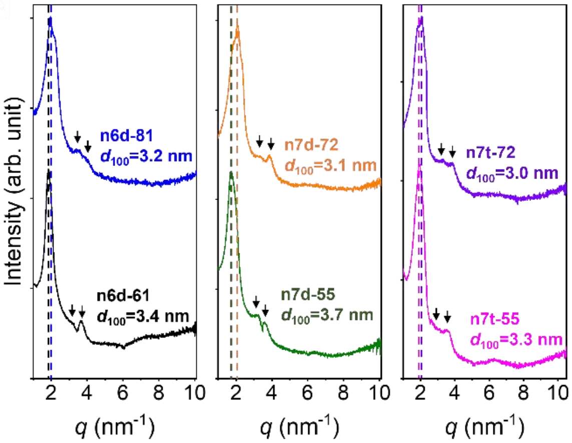

D

High ε solvent (Anions in solvent continuum)					
Mesophase	Cylinder fraction (% by volume)	*d*-spacing (nm)	$2r_f$ (nm)	**s_x (nm)**	2δ (nm)
n6d-81	67.6	3.2	3.19	**0.506**	1.08
n5t-76	64.4	3.1	3.02	**0.563**	1.12
n7t-72	61.5	3.0	2.85	**0.611**	1.15
n7d-72	61.1	3.1	2.94	**0.641**	1.20
n6t-61	53.3	3.2	2.83	**0.862**	1.43
n6d-61	52.9	3.4	3.00	**0.926**	1.53
n7t-55	48.7	3.3	2.79	**1.02**	1.61
n7d-55	48.4	3.7	3.12	**1.15**	1.81

Fig. S6. GISAXS data and transport limiting dimension analysis for additional mesophases. The thin-films were prepared through spin-coating the mesophase solution atop the silicon substrates, followed by photoinitiated polymerization. (**A**) Tabulated mesophase formula prepared for the structural characterization. (**B**) 2D GISAXS images of polymerized mesophases indicate the H_I was retained, and the cylinders remained in-plane morphology. (**C**) 1D SAXS data demonstrate the tunability in the center-to-center distance defined by H_I mesophase compositions. A consistent spacing ratio 1: $\sqrt{3}$: $\sqrt{4}$ is followed. (**D**) Tabulated data of the critical dimensions. The s_x column represents the transport limiting dimension assuming an in-plane cylinder orientation.

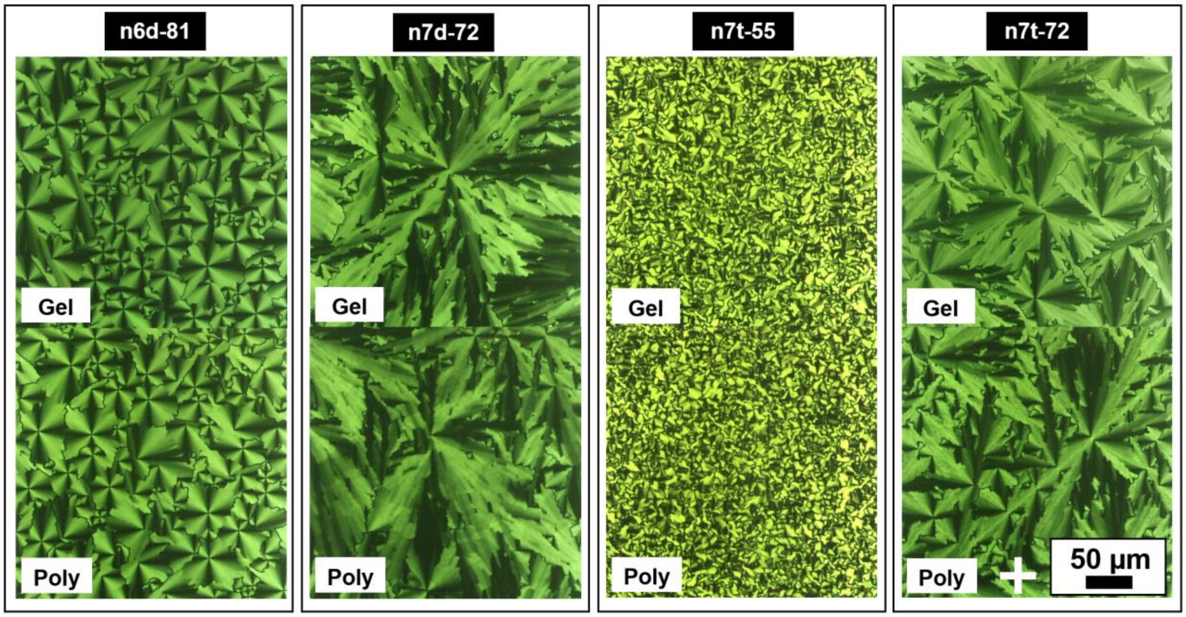

Fig. S7. High magnification POM images from additional prepared mesophases. Thin films were prepared from spin-coating the solution on pre-cleaned glass slides, followed by gentle heating to develop the birefringence domains. Micrographs were recorded before and after the photoinitiated cross-linking. Albeit the difference in compositions from their original formulas, consistent focal conic textures were identified before and after the polymerization

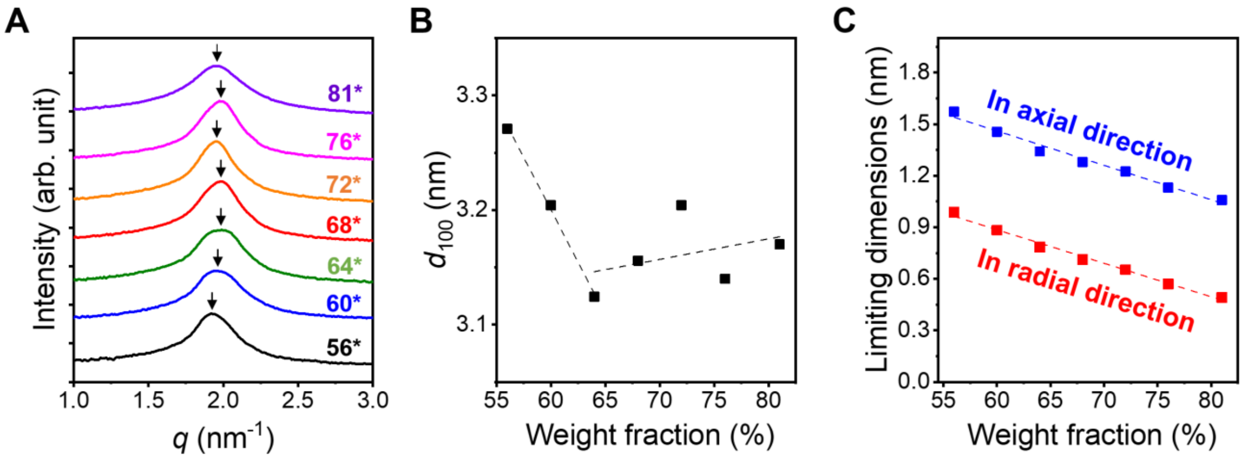

Fig. S8. 1D GISAXS data of the n5t mesophases with different surfactant concentrations and the associated limiting dimension analysis. Mesophases were cast on PVP modified substrates. (**A**) Magnified 1D integrated data of the n5t with a consistent cross-linker concentration, but small increments in weight fractions from 56 to 81 wt.%. (**B**) The change in d_{100} possesses a non-linear relationship to weight fractions. The non-monotonic correlation may have resulted from the interplay between cross-linker swelling and surfactant packing in the lyotropic phase. (**C**) A survey of limiting dimensions reveals the confined limiting dimensions are linearly related to surfactant weight fractions with precise increments ~0.1 nm. The limiting dimension in the radial direction is linear in the d-spacing and varies as the square root of the volume fraction. Thus, at higher weight fractions, the modest increase in the d-spacing ~3% is offset by the larger decrease in the square root of the volume fraction term $(4/3)^{1/2} - (8\varphi/\sqrt{3}\pi)^{1/2}$ ~40%. As a result, the transport limiting dimension continues to decrease with volume fraction, despite the increase in the d-spacing.

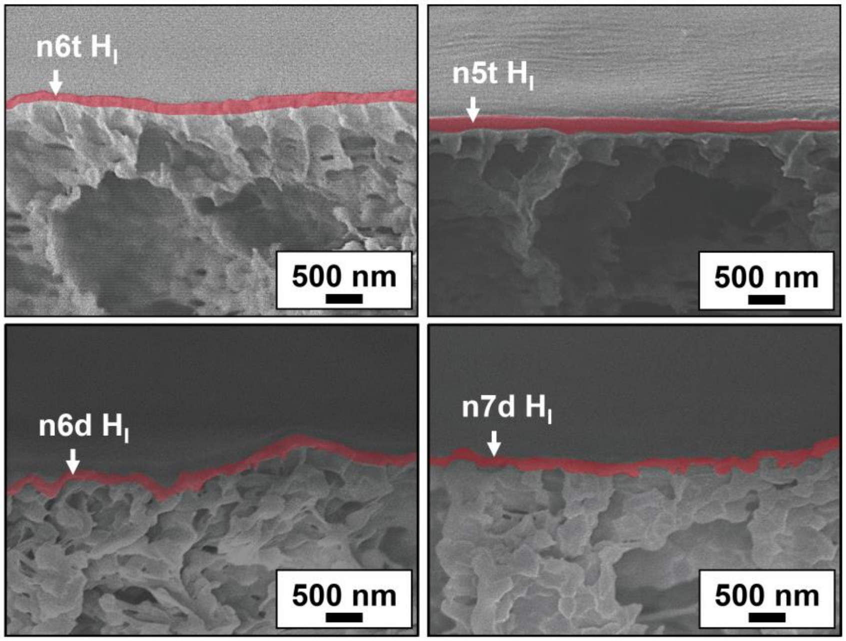

Fig. S9. Magnified micrographs of the thin-film composite membranes. The color-overlay represents the film thickness of the self-assembled thin membrane atop the PVDF ultrafiltration support.

A

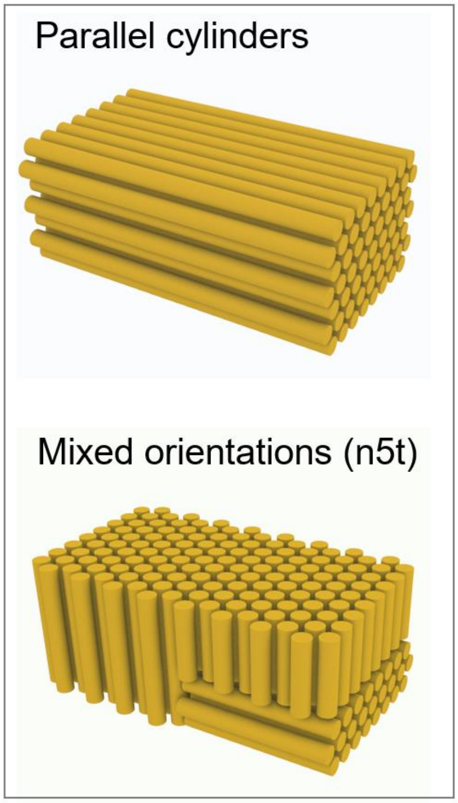

B

Mesophase	Estimated P ($L\ m^{-2}\ h^{-1}\ bar^{-1}\ \mu m$)
n5t	3.0
n6t	2.3
n6d	2.5
n7d	4.9

C

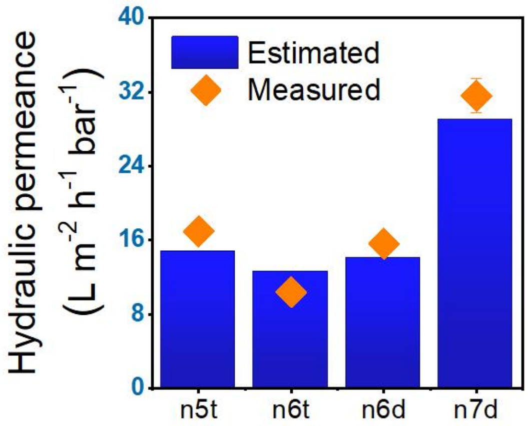

Fig. S10. Estimating hydraulic permeance from different nanostructures from theory. (**A**) Here n6t, n6d, and n7d are modeled with a parallel orientation, and the n5t is considered to possess mixed cylinder orientations mostly in perpendicular cylinders. For n5t, parallel cylinders are assumed to project half of the surface area, and span across half of the film thickness. (**B**) The thickness normalized hydraulic permeabilities were calculated using the results from the structural analysis, plus assuming a consistent tortuosity ~2.5 for each membrane. (**C**) The calculated hydraulic permeances are similar to the measured permeances.

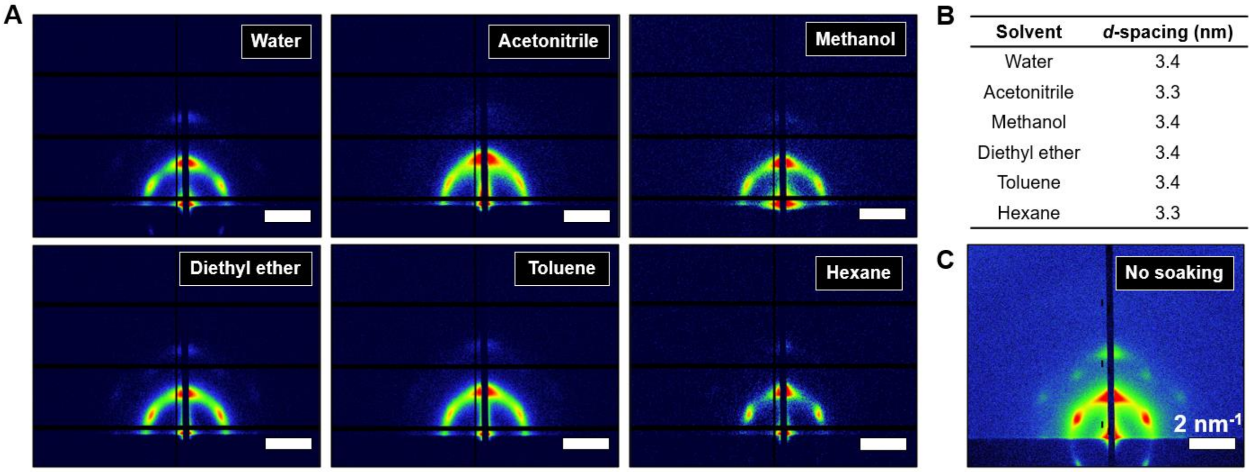

Solvent	*d*-spacing (nm)
Water	3.4
Acetonitrile	3.3
Methanol	3.4
Diethyl ether	3.4
Toluene	3.4
Hexane	3.3

Fig. S11. A survey of 2D GISAXS patterns for the cross-linked n6d mesophases immersed in different organic solvents for a pre-determined amount of time. After the prolonged soaking period, films were dipped on towels and dried in high-vacuum prior to imaging. A consistent d_{100} spacing ~3.4-nm was observed based on the 1D-integrated data, and there was no significant nanostructural disruption during this process.

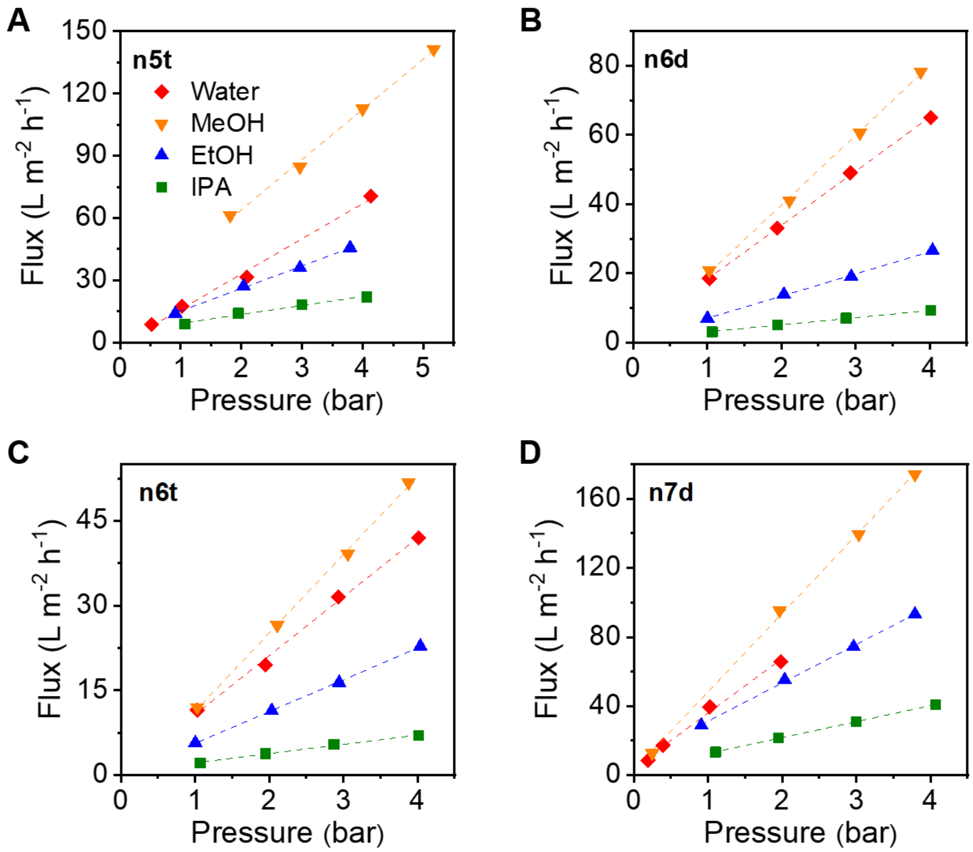

Fig. S12. Solvent flux as a function of transmembrane pressure for different H_I membranes. Each membrane is listed with four solvents, including water (labeled as red diamond), methanol (labeled as orange triangle), ethanol (labeled as blue triangle) and isopropanol (labeled as green square). The solvent permeance is derived from the linear regression as represented by the dashed line.

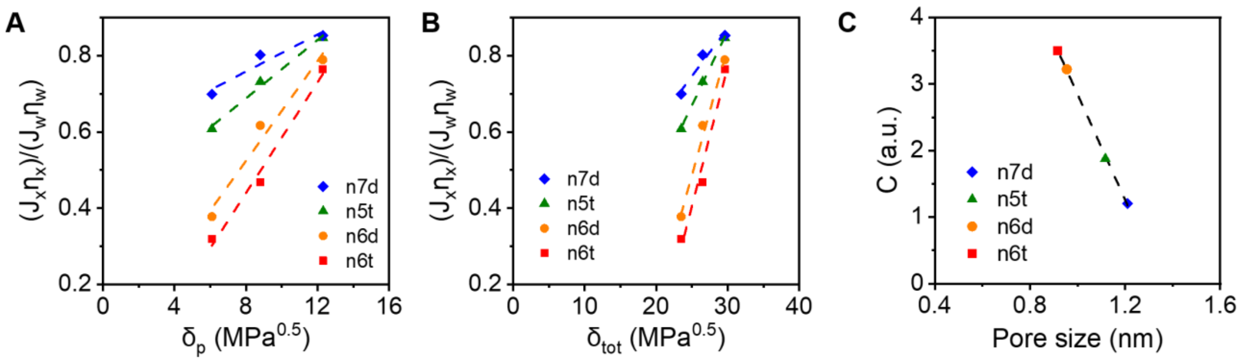

D

Solvents	d_m (nm)	Viscosity at 20 ℃ (mPa s)	δ_p (Mpa$^{1/2}$)	δ_{tot} (Mpa$^{1/2}$)
Water	0.39	1	16	47.8
Methanol	0.50	0.59	12.3	29.6
Ethanol	0.57	1.1	8.8	26.5
Isopropanol	0.62	2.4	6.1	23.5
Hexane	0.75	0.31	0	14.9

Fig. S13. The product of solvent permeance and viscosity normalized using the water value. The product is listed as a function of (**A**) the polarity factor in the Hansen solubility parameter and (**B**) the total Hansen solubility parameter for different H_I membranes. A linear correlation is established. (**C**) A recreation of the Buekenhoudt flux model suggests the water flux normalized proportionality factor is linear with pore size. The calculations were based on the Hansen solubility parameters and the physical properties of the organic solvents tabulated in (**D**), data retrieved from literature (*13*, *48*, *57*).

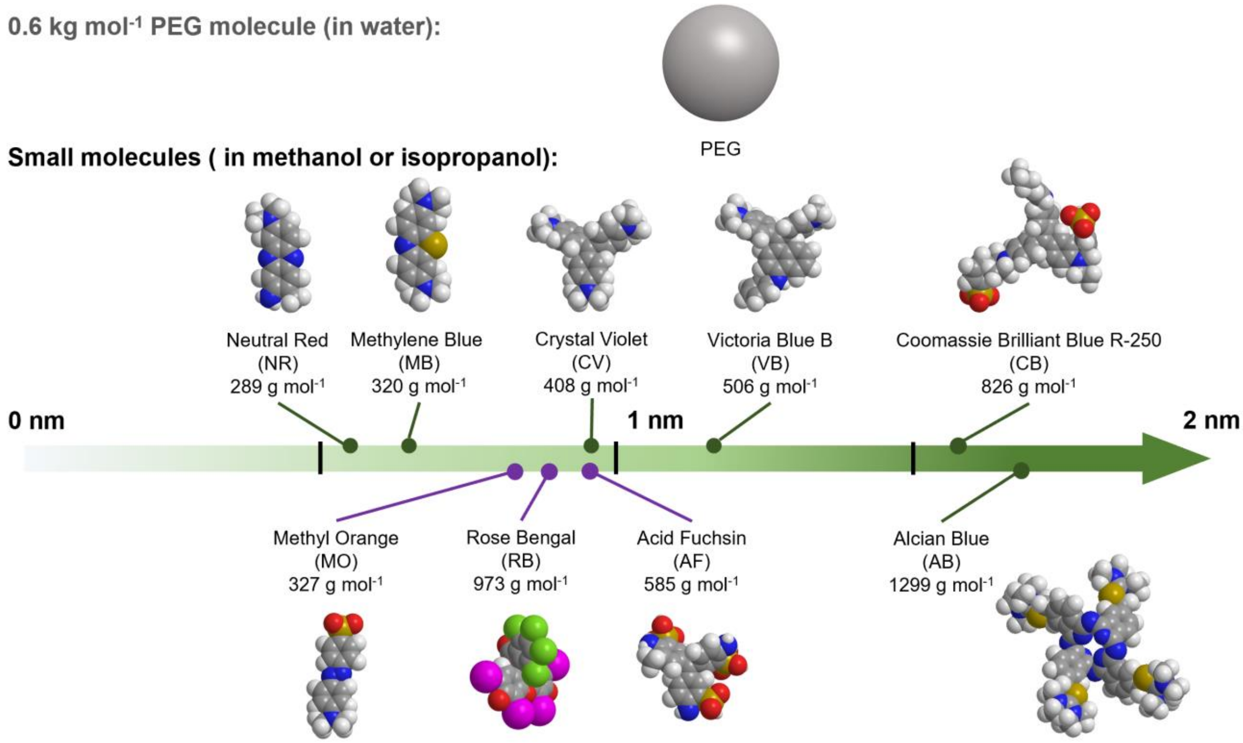

Fig. S14. Schematic of spacing filling models for solutes involved in the solute separation experiments with arrows linking to the corresponding solute diameter. The geometric mean diameters are derived from the molecular dimensions estimated by the Chem3D software package for dye molecules, or directly retrieved from literature for PEG600.

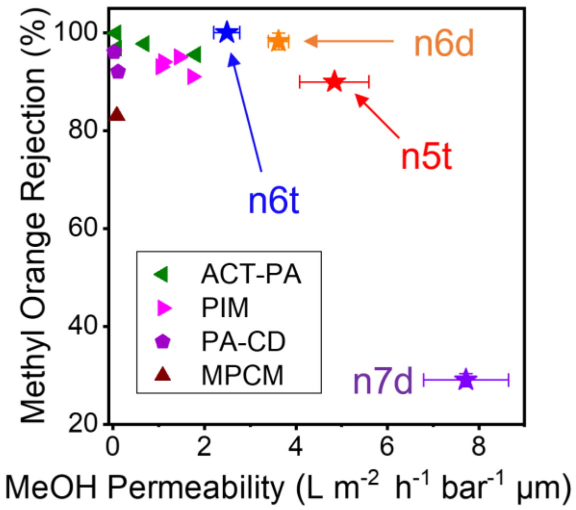

Fig. S15. Methanol permeability and methyl orange rejection performance of the H_I membranes. Membrane performance is listed along with literature data. Here the self-assembled membranes stand out with superior solvent permeability relative to amorphous materials at high solute rejections. Error bars correspond to 95% confidence interval from multiple measurements.

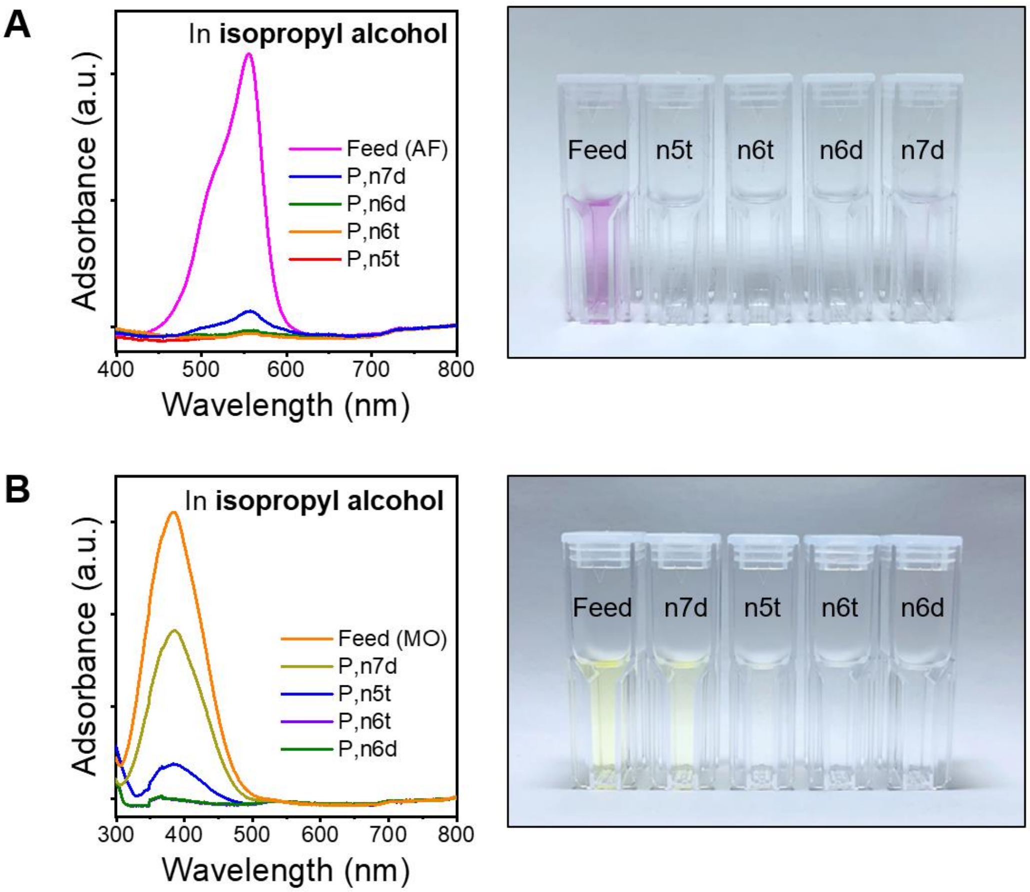

Fig. S16. UV-visible (UV-vis) spectra and photographs of the permeate collected from isopropyl alcohol (IPA) solution. The data are showing the solute separation of acid fuchsin (AF) and methyl orange (MO) permeate from the H_I membranes in IPA. Note the n7d membrane, the H_I with the largest theoretical pore size almost completely rejected AF (~590 g mol^{-1}). Photo credit: Yizhou Zhang, University of Pennsylvania.

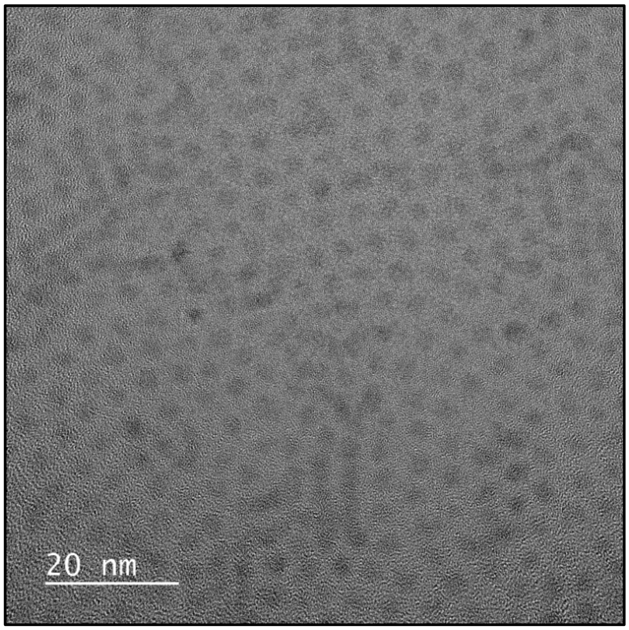

Fig. S17. Transmission electron micrograph for the CdSe nanoparticle reveals a spherical solute geometry with a diameter of 2.7-nm.

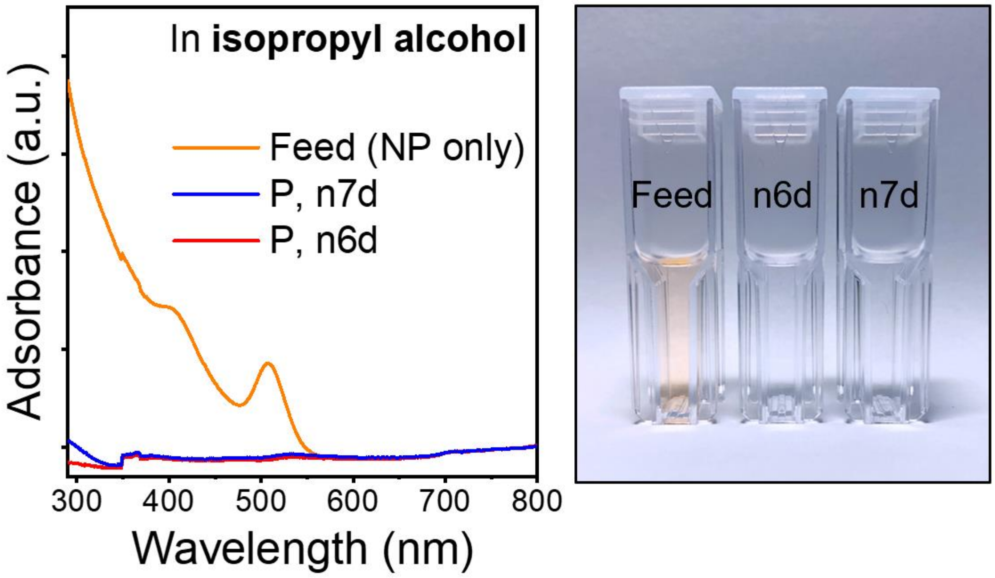

Fig. S18. UV-vis spectra and photographs demonstrating the complete solute separation of the CdSe nanoparticle dissolved in IPA by n6d and n7d membranes. Photo credit: Yizhou Zhang, University of Pennsylvania.

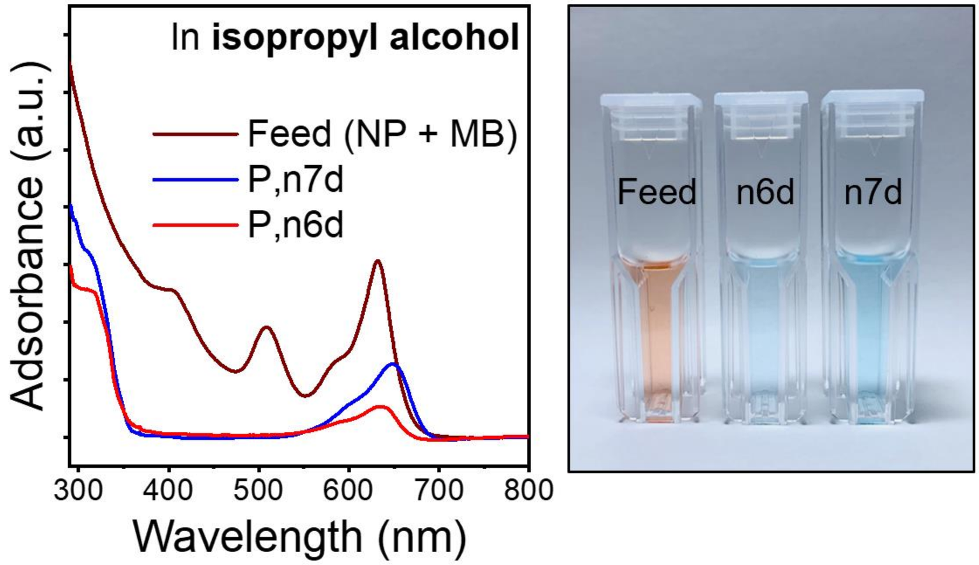

Fig. S19. UV-vis spectra and photographs showing the competitive solute separation of the CdSe nanoparticle and methylene blue (MB) dissolved in IPA by n6d and n7d H_I membranes. Photo credit: Yizhou Zhang, University of Pennsylvania.

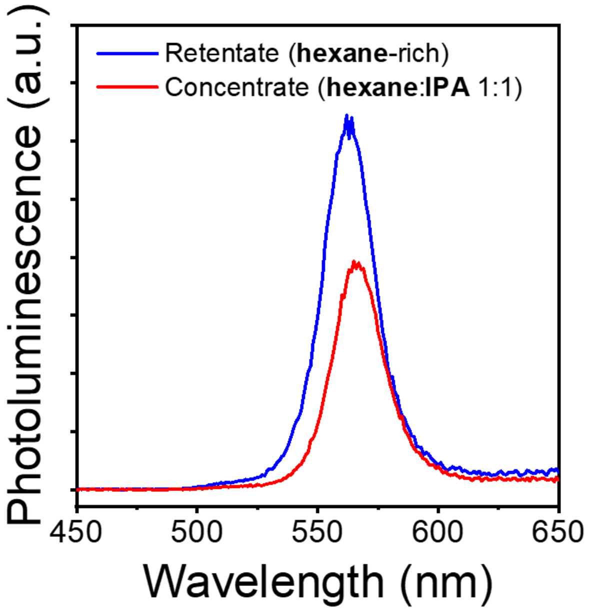

Fig. S20. An enhancement in the photoluminescence intensity is observed after the rejection of CdSe nanoparticle dispersed in an equal volume of IPA and hexane by a n6d membrane. The solution was normalized to the same nanoparticle concentration. While the nanoparticle is not fluorescent in IPA solution, the enhancement may evidence a potential solvent exchange behavior with enriched hexane in the feed.

REFERENCES AND NOTES

1. R. P. Lively, D. S. Sholl, From water to organics in membrane separations. *Nat. Mater.* **16**, 276–279 (2017).

2. D. S. Sholl, R. P. Lively, Seven chemical separations to change the world. *Nature* **532**, 435–437 (2016).

3. K. A. Thompson, R. Mathias, D. Kim, J. Kim, N. Rangnekar, J. R. Johnson, S. J. Hoy, I. Bechis, A. Tarzia, K. E. Jelfs, B. A. M.c Cool, A. G. Livingston, R. P. Lively, M. G. Finn, N-aryl–linked spirocyclic polymers for membrane separations of complex hydrocarbon mixtures. *Science* **369**, 310–315 (2020).

4. P. Marchetti, M. F. Jimenez Solomon, G. Szekely, A. G. Livingston, Molecular separation with organic solvent nanofiltration: A critical review. *Chem. Rev.* **114**, 10735–10806 (2014).

5. C. Liu, G. Dong, T. Tsuru, H. Matsuyama, Organic solvent reverse osmosis membranes for organic liquid mixture separation: A review. *J. Membr. Sci.* **620**, 118882 (2021).

6. M. Galizia, K. P. Bye, Advances in organic solvent nanofiltration rely on physical chemistry and polymer chemistry. *Front. Chem.* **6**, 511 (2018).

7. S. Hermans, H. Mariën, C. Van Goethem, I. F. Vankelecom, Recent developments in thin film (nano)composite membranes for solvent resistant nanofiltration. *Curr. Opin. Chem. Eng.* **8**, 45–54 (2015).

8. H. B. Park, J. Kamcev, L. M. Robeson, M. Elimelech, B. D. Freeman, Maximizing the right stuff: The trade-off between membrane permeability and selectivity. *Science* **356**, eaab0530 (2017).

9. D. L. Gin, R. D. Noble, Designing the next generation of chemical separation membranes. *Science* **332**, 674–676 (2011).

10. V. Freger, Nanoscale heterogeneity of polyamide membranes formed by interfacial polymerization. *Langmuir* **19**, 4791–4797 (2003).

11. A. W. Mohammad, Y. H. Teow, W. L. Ang, Y. T. Chung, D. L. Oatley-Radcliffe, N. Hilal, Nanofiltration membranes review: Recent advances and future prospects. *Desalination* **356**, 226–254 (2015).

12. J. Luo, Y. Wan, Effects of pH and salt on nanofiltration—A critical review. *J. Membr. Sci.* **438**, 18–28 (2013).

13. S. Karan, Z. Jiang, A. G. Livingston, Sub–10 nm polyamide nanofilms with ultrafast solvent transport for molecular separation. *Science* **348**, 1347–1351 (2015).

14. P. Gorgojo, S. Karan, H. C. Wong, M. F. Jimenez-Solomon, J. T. Cabral, A. G. Livingston, Ultrathin polymer films with intrinsic microporosity: Anomalous solvent permeation and high flux membranes. *Adv. Funct. Mater.* **24**, 4729–4737 (2014).

15. M. F. Jimenez-Solomon, Q. Song, K. E. Jelfs, M. Munoz-Ibanez, A. G. Livingston, Polymer nanofilms with enhanced microporosity by interfacial polymerization. *Nat. Mater.* **15**, 760–767 (2016).

16. B. Liang, H. Wang, X. Shi, B. Shen, X. He, Z. A. Ghazi, N. A. Khan, H. Sin, A. M. Khattak, L. Li, Z. Tang, Microporous membranes comprising conjugated polymers with rigid backbones enable ultrafast organic-solvent nanofiltration. *Nat. Chem.* **10**, 961–967 (2018).

17. S. Kandambeth, B. P. Biswal, H. D. Chaudhari, K. C. Rout, Shebeeb Kunjattu H., S. Mitra, S. Karak, A. Das, R. Mukherjee, U. K. Kharul, R. Banerjee, Selective molecular sieving in self-standing porous covalent-organic-framework membranes. *Adv. Mater.* **29**, 1603945 (2017).

18. D. B. Shinde, G. Sheng, X. Li, M. Ostwal, A.-H. Emwas, K.-W. Huang, Z. Lai, Crystalline 2D covalent organic framework membranes for high-flux organic solvent nanofiltration. *J. Am. Chem. Soc.* **140**, 14342–14349 (2018).

19. E. Barankova, X. Tan, L. F. Villalobos, E. Litwiller, K. V. Peinemann, A metal chelating porous polymeric support: The missing link for a defect-free metal–organic framework composite membrane. *Angew. Chem. Int. Ed.* **56**, 2965–2968 (2017).

20. J. R. Werber, C. O. Osuji, M. Elimelech, Materials for next-generation desalination and water purification membranes. *Nat. Rev. Mater.* **1**, 16018 (2016).

21. D. L. Gin, X. Lu, P. R. Nemade, C. S. Pecinovsky, Y. Xu, M. Zhou, Recent advances in the design of polymerizable lyotropic liquid-crystal assemblies for heterogeneous catalysis and selective separations. *Adv. Funct. Mater.* **16**, 865–878 (2006).

22. T. Kato, J. Uchida, T. Ichikawa, T. Sakamoto, Functional liquid crystals towards the next generation of materials. *Angew. Chem. Int. Ed.* **57**, 4355–4371 (2018).

23. X. Feng, M. E. Tousley, M. G. Cowan, B. R. Wiesenauer, S. Nejati, Y. Choo, R. D. Noble, M. Elimelech, D. L. Gin, C. O. Osuji, Scalable fabrication of polymer membranes with vertically aligned 1 nm pores by magnetic field directed self-assembly. *ACS Nano* **8**, 11977–11986 (2014).

24. T. Ichikawa, T. Kato, H. Ohno, 3D continuous water nanosheet as a gyroid minimal surface formed by bicontinuous cubic liquid-crystalline zwitterions. *J. Am. Chem. Soc.* **134**, 11354–11357 (2012).

25. T. Sakamoto, T. Ogawa, H. Nada, K. Nakatsuji, M. Mitani, B. Soberats, K. Kawata, M. Yoshio, H. Tomioka, T. Sasaki, M. Kimura, M. Henmi, T. Kato, Development of nanostructured water treatment membranes based on thermotropic liquid crystals: Molecular design of sub-nanoporous materials. *Adv. Sci.* **5**, 1700405 (2018).

26. X. Feng, S. Nejati, M. G. Cowan, M. E. Tousley, B. R. Wiesenauer, R. D. Noble, M. Elimelech, D. L. Gin, C. O. Osuji, Thin polymer films with continuous vertically aligned 1 nm pores fabricated by soft confinement. *ACS Nano* **10**, 150–158 (2016).

27. O. Q. Imran, N. K. Kim, L. N. Bodkin, G. E. Dwulet, X. Feng, K. Kawabata, M. Elimelech, D. L. Gin, C. O. Osuji, Nanoscale thickness control of nanoporous films derived from directionally photopolymerized mesophases. *Adv. Mater. Interfaces* **8**, 2001977 (2021).

28. S. M. Dischinger, J. Rosenblum, R. D. Noble, D. L. Gin, Evaluation of a nanoporous lyotropic liquid crystal polymer membrane for the treatment of hydraulic fracturing produced water via cross-flow filtration. *J. Membr. Sci.* **592**, 117313 (2019).

29. J. E. Bara, A. K. Kaminski, R. D. Noble, D. L. Gin, Influence of nanostructure on light gas separations in cross-linked lyotropic liquid crystal membranes. *J. Membr. Sci.* **288**, 13–19 (2007).

30. M. Zhou, P. R. Nemade, X. Lu, X. Zeng, E. S. Hatakeyama, R. D. Noble, D. L. Gin, New type of membrane material for water desalination based on a cross-linked bicontinuous cubic lyotropic liquid crystal assembly. *J. Am. Chem. Soc.* **129**, 9574–9575 (2007).

31. B. M. Carter, B. R. Wiesenauer, E. S. Hatakeyama, J. L. Barton, R. D. Noble, D. L. Gin, Glycerol-based bicontinuous cubic lyotropic liquid crystal monomer system for the fabrication of thin-film membranes with uniform nanopores. *Chem. Mater.* **24**, 4005–4007 (2012).

32. C. R. Kasprzak, E. T. Scherzinger, A. Sarkar, M. Miao, D. H. Porcincula, A. M. Madriz, Z. M. Pennewell, S. S. Chau, R. Fernando, M. Stefik, S. Zhang, Ordered nanostructures of carbon nanotube–polymer composites from lyotropic liquid crystal templating. *Macromol. Chem. Phys.* **219**, 1800197 (2018).

33. M. A. DePierro, C. A. Guymon, Polymer structure development in lyotropic liquid crystalline solutions. *Macromolecules* **47**, 5728–5738 (2014).

34. G. P. Sorenson, K. L. Coppage, M. K. Mahanthappa, Unusually stable aqueous lyotropic gyroid phases from gemini dicarboxylate surfactants. *J. Am. Chem. Soc.* **133**, 14928–14931 (2011).

35. X. Feng, K. Kawabata, G. Kaufman, M. Elimelech, C. O. Osuji, Highly selective vertically aligned nanopores in sustainably derived polymer membranes by molecular templating. *ACS Nano* **11**, 3911–3921 (2017).

36. M. E. Tousley, X. Feng, M. Elimelech, C. O. Osuji, Aligned nanostructured polymers by magnetic-field-directed self-assembly of a polymerizable lyotropic mesophase. *ACS Appl. Mater. Interfaces* **6**, 19710–19717 (2014).

37. X. Feng, K. Kawabata, M. G. Cowan, G. E. Dwulet, K. Toth, L. Sixdenier, A. Haji-Akbari, R. D. Noble, M. Elimelech, D. L. Gin, C. O. Osuji, Single crystal texture by directed molecular self-assembly along dual axes. *Nat. Mater.* **18**, 1235–1243 (2019).

38. X. Feng, Q. Imran, Y. Zhang, L. Sixdenier, X. Lu, G. Kaufman, U. Gabinet, K. Kawabata, M. Elimelech, C. O. Osuji, Precise nanofiltration in a fouling-resistant self-assembled membrane with water-continuous transport pathways. *Sci. Adv*. **5**, eaav9308 (2019).

39. Y. Zhang, R. Dong, U. R. Gabinet, R. Poling-Skutvik, N. K. Kim, C. Lee, O. Q. Imran, X. Feng, C. O. Osuji, Rapid fabrication by lyotropic self-assembly of thin nanofiltration membranes with uniform 1 nanometer pores. *ACS Nano* **15**, 8192–8203 (2021).

40. W. A. Phillip, M. A. Hillmyer, E. L. Cussler, Cylinder orientation mechanism in block copolymer thin films upon solvent evaporation. *Macromolecules* **43**, 7763–7770 (2010).

41. C. Sinturel, M. Vayer, M. Morris, M. A. Hillmyer, Solvent vapor annealing of block polymer thin films. *Macromolecules* **46**, 5399–5415 (2013).

42. A. Faraone, S. Magazu, G. Maisano, P. Migliardo, E. Tettamanti, V. Villari, The puzzle of poly (ethylene oxide) aggregation in water: Experimental findings. *J. Chem. Phys.* **110**, 1801–1806 (1999).

43. M. Meireles, A. Bessieres, I. Rogissart, P. Aimar, V. Sanchez, An appropriate molecular size parameter for porous membranes calibration. *J. Membr. Sci.* **103**, 105–115 (1995).

44. J. Shao, R. E. Baltus, Hindered diffusion of dextran and polyethylene glycol in porous membranes. *AIChE J.* **46**, 1149–1156 (2000).

45. M. Sobera, C. Kleijn, Hydraulic permeability of ordered and disordered single-layer arrays of cylinders. *Phys. Rev. E* **74**, 036301 (2006).

46. J. Drummond, M. Tahir, Laminar viscous flow through regular arrays of parallel solid cylinders. *Int. J. Multiphase Flow* **10**, 515–540 (1984).

47. L. J. Zeman, A. Zydney, *Microfiltration and Ultrafiltration: Principles and Applications* (Marcel Dekker, 1996).

48. A. Buekenhoudt, F. Bisignano, G. De Luca, P. Vandezande, M. Wouters, K. Verhulst, Unravelling the solvent flux behaviour of ceramic nanofiltration and ultrafiltration membranes. *J. Membr. Sci.* **439**, 36–47 (2013).

49. B. Guzelturk, O. Erdem, M. Olutas, Y. Kelestemur, H. V. Demir, Stacking in colloidal nanoplatelets: Tuning excitonic properties. *ACS Nano* **8**, 12524–12533 (2014).

50. J. Chen, J. Song, X. Sun, W. Deng, C. Jiang, W. Lei, J. Huang, R. Liu, An oleic acid-capped CdSe quantum-dot sensitized solar cell. *Appl. Phys. Lett.* **94**, 153115 (2009).

51. S. Sorribas, P. Gorgojo, C. Téllez, J. Coronas, A. G. Livingston, High flux thin film nanocomposite membranes based on metal–organic frameworks for organic solvent nanofiltration. *J. Am. Chem. Soc.* **135**, 15201–15208 (2013).

52. L. F. Villalobos, T. Huang, K. V. Peinemann, Cyclodextrin films with fast solvent transport and shape-selective permeability. *Adv. Mater.* **29**, 1606641 (2017).

53. J. Liu, D. Hua, Y. Zhang, S. Japip, T. S. Chung, Precise molecular sieving architectures with Janus pathways for both polar and nonpolar molecules. *Adv. Mater.* **30**, 1705933 (2018).

54. T. Huang, T. Puspasari, S. P. Nunes, K. V. Peinemann, Ultrathin 2D-layered cyclodextrin membranes for high-performance organic solvent nanofiltration. *Adv. Funct. Mater.* **30**, 1906797 (2020).

55. T. Huang, B. A. Moosa, P. Hoang, J. Liu, S. Chisca, G. Zhang, M. AlYami, N. M. Khashab, S. P. Nunes, Molecularly-porous ultrathin membranes for highly selective organic solvent nanofiltration. *Nat. Commun.* **11**, 5822 (2020).

56. Y.-x. Shen, W. Song, D. R.yan Barden, T. Ren, C. Lang, H. Feroz, C. B. Henderson, P. O. Saboe, D. Tsai, H. Yan, P. J. Butler, G. C. Bazan, W. A. Phillip, R. J. Hickey, P. S. Cremer, H. Vashisth, M. Kumar, Achieving high permeability and enhanced selectivity for Angstrom-scale separations using artificial water channel membranes. *Nat. Commun.* **9**, 2294 (2018).

57. B. Van der Bruggen, J. Schaep, D. Wilms, C. Vandecasteele, Influence of molecular size, polarity and charge on the retention of organic molecules by nanofiltration. *J. Membr. Sci.* **156**, 29–41 (1999).
